# METTL14 Regulates the Expression of Genes Related to Interferon, Interleukin and MHC Class I in Nasopharyngeal Carcinoma Cells

**DOI:** 10.1002/cam4.71371

**Published:** 2025-11-28

**Authors:** Zhihao Zhou, Jing Wang, Lingjun Shen, Liuxin Han, Qiwen Li, Aibing Wu, Jing Li, Zuming Liang, Litong Zhu, Danhua He, Ying Zhou, Shihao Huang, Zhanlin Zhao, Jinge Cong, Zhitao Peng, Ping Zhao, Shuna Ye, Binyi Bai, Xuanjia Hong, Guanqi Dai, Ye Lei, Wentao Zhao, Junshuang Jia, Xiaolin Lin, Dong Xiao, Yuqin Zhang, Taoyan Lin

**Affiliations:** ^1^ Laboratory Animal Management Center, Cancer Research Institute, School of Basic Medical Sciences Southern Medical University Guangzhou China; ^2^ Department of Tuberculosis Yunnan Clinical Medical Center for Infectious Diseases, the Third People's Hospital of Kunming (The Sixth Affiliated Hospital of Dali University) Kunming China; ^3^ Department of Critical Care Medicine Blood Purification Centre, the Third People's Hospital of Kunming (Yunnan Infectious Diseases Clinical Medical Center, the Sixth Affiliated Hospital of Dali University) Kunming China; ^4^ Department of Oncology The Central People's Hospital of Zhanjiang Zhanjiang China; ^5^ Radiotherapy Center, the First People's Hospital of Chenzhou Chenzhou China; ^6^ State Key Laboratory of Organ Failure Research, National Clinical Research Center for Kidney Disease, Guangdong Provincial Institute of Nephrology, Guangdong Provincial Key Laboratory of Renal Failure Research, Division of Nephrology, Nanfang Hospital Southern Medical University Guangzhou China; ^7^ Department of Gastrointestinal Oncology Peking University Cancer Hospital Yunnan (Yunnan Cancer Hospital, the Third Affiliated Hospital of Kunming Medical University) Kunming China; ^8^ Department of Radiation Oncology, Nanfang Hospital Southern Medical University Guangzhou China; ^9^ Department of Pharmacy, Nanfang Hospital Southern Medical University Guangzhou China

**Keywords:** immune and inflammation‐related genes, interferon‐induced genes, interleukins‐related genes, METTL14, MHC class I genes, nasopharyngeal carcinoma, TNF‐related genes

## Abstract

**Background:**

Nasopharyngeal carcinoma (NPC), highly prevalent in southern China, often leads to treatment failure in advanced stages due to recurrence or metastasis. While METTL14 plays a crucial role in cancer, its regulation of immune‐ and inflammation‐related genes remains poorly understood. This study aims to investigate whether METTL14 is involved in regulating the expression of genes associated with tumor necrosis factor (TNF), interferon (IFN), interleukin (IL), and MHC class I in NPC cells.

**Methods:**

Quantitative real‐time polymerase chain reaction (qRT‐PCR) and Western blotting were used to examine METTL14 expression in NPC cells with either shMETTL14 knockdown or METTL14 overexpression. RNA sequencing was performed to identify differentially expressed genes associated with TNF, IFN, IL, and MHC class I in NPC cells, and qRT‐PCR was used to validate the expression of these genes. A cytokine production assay was conducted to measure the levels of TNFSF9, IFI16, IL‐6, IL‐7, CXCL10, and HLA‐B.

**Results:**

Our study demonstrates that METTL14 potentially plays a fundamental role in modulating the expression of genes associated with TNF, IFN, IL, and MHC class I in NPC cells. RNA sequencing of NPC cells showed that METTL14 upregulated or downregulated several genes associated with immunity and inflammation, including TNF, IFN, IL, and MHC class I. RNA interference‐mediated knockdown of METTL14 revealed its regulatory effects on TNF‐related genes (e.g., TNFRSF12A, TNFRSF9, TNFSF15), IFN‐induced genes (e.g., PSMC6, PSMD1, PSMA2, PSMA3, PSMB2), and IL‐related genes (e.g., CCL5, IL1B, IL32, IL7R, IL6, IL7, CXCL2). Furthermore, METTL14 was found to positively regulate MHC class I gene expression (e.g., HLA‐A, ‐B, ‐C, ‐E, ‐F) in NPC cells.

**Conclusions:**

These findings demonstrate, for the first time, that METTL14 modulates the expression of genes related to TNF, IFN, IL, and MHC class I in NPC, proposing a novel role for METTL14 in linking inflammation with cancer, a function that has not been fully elucidated.

## 1 Introduction

Nasopharyngeal carcinoma (NPC) is a prevalent malignant tumor in southern China, characterized by insidious onset and high malignancy, often presenting with cervical lymph node metastasis [1] [2]. Despite improvements in diagnostic and treatment methods, around 20%–30% of patients with advanced‐stage NPC still experience treatment failure due to recurrence and/or metastasis [1, 2, 3, 4]. Hence, elucidating the molecular mechanisms underlying NPC progression is essential for developing effective strategies to address recurrence and metastasis.

Clearly, epigenetic modifications are pivotal in tumorigenesis, and among them, m^6^A methylation of RNA is a major and highly abundant posttranscriptional modification of mRNA in eukaryotic organisms [5, 6]. The m^6^A modification is mediated by a group of enzymes known as “writers,” primarily consisting of methyltransferase‐like 3 (METTL3) [7], METTL14 [8], METTL16 [9], and Wilms tumor 1‐associating protein (WTAP) [10], among others. METTL14 is central to various common cancers, including hepatocellular carcinoma (HCC) [11], breast cancer (BC) [12], prostate cancer [13], glioma [14], and leukemia [15], nonsmall cell lung cancer (NSCLC) [16]. Recently, extensive research has highlighted the prominent role of METTL14 in NPC, involving lipid metabolism [17] [18], proliferation [19], invasion, and metastasis [20]. Specifically, METTL14 enhances lipid metabolism reprogramming and sustains the progression of NPC [17]; METTL14 may enhance NPC cell proliferation by modulating the stability of AOC1 mRNA [19]. Additionally, METTL3, METTL14, and WTAP synergistically facilitate NPC invasion and metastasis and induce radioresistance in NPC cells through DNA repair mechanisms [20].

Our investigation into METTL14's role in EMT, migration, and invasion in NPC unexpectedly revealed its significant regulatory influence on inflammatory and immune‐related genes through RNA‐seq analysis. Specifically, METTL14 modulates IFN‐related genes (IFI27, IRS1, PSMA2, PSMA3), IL‐associated genes (CCL5, IL1B, IL32), and MHC class I genes (HLA‐A, B, C), implying a potential mechanistic link between METTL14 and cancer‐associated inflammatory responses. Extensive research has revealed that METTL14 functions as a central mediator in modulating inflammatory responses across various diseases, such as coronary artery disease [21, 22], psoriasis [23, 24], rheumatoid arthritis [25], glomerulonephritis [26], colitis [27], and ankylosing spondylitis [28]. Mechanistically, METTL14 enhances endothelial inflammation by increasing m^6^A modification of FOXO1 [21] and modulates macrophage inflammatory responses through the NF‐κB/IL‐6 signaling pathway [22]. In psoriasis, METTL14 interacts with UCA1 to regulate the HIF‐1α/NF‐κB axis [23], while also modulating the SOCS3/STAT3 pathway to attenuate IL‐6‐mediated inflammation [24]. Furthermore, METTL14 catalyzes m^6^A methylation of TNFAIP3 in rheumatoid arthritis [25], promotes glomerulopathy progression through Sirt1 suppression [28], and stabilizes NF‐κB mRNA to regulate colitis [27]. Additionally, METTL14 is involved in regulating TNF‐related genes [21] [29], IFN‐related pathways in tumors [30], IL‐6‐induced inflammation [22], and MHC class I‐mediated tumor immune escape [31, 32]. Our findings are in strong agreement with existing literature and highlight the emerging role of METTL14 in linking inflammation and cancer, although this role requires further investigation. These observations prompt us to explore whether METTL14 is engaged in regulating the expression of genes associated with TNF, IFN, IL, and MHC class I in NPC cells.

## 2 Materials and Methods

### 2.1 Cell Culture

Human immortalized nasopharyngeal epithelial cells (NP69) and various human NPC cell lines, including CNE2, SUNE1, S18, 5‐8F, C666‐1 (EBV‐positive cell line), HK1, HK1‐EBV, HONE1 and HONE1‐EBV, were kindly provided by Prof. S.‐W. Tsao, Prof. Qiao Tao, Prof. Yixin Zeng, Prof. Musheng Zeng, and Dr. Dengke Li, as previously described in published works [33]. The NPC cell lines were cultured in RPMI 1640 medium containing 10% fetal bovine serum (FBS; PAN Biotech, Cegrogen Biotech) and maintained under standard conditions at 37°C with 5% CO2. The NP69 cell line was grown in keratinocyte/serum‐free medium (Invitrogen), while HEK293T cells were cultured in DMEM medium containing 10% FBS (VivaCell Biotech) in the same incubator conditions. The authenticity of all cell lines was confirmed by short tandem repeat (STR) fingerprinting conducted by GUANGZHOU IGE BIOTECHNOLOGY Co. Ltd. (Guangzhou, China).

### 2.2 Plasmids, Lentivirus Production and Transduction

The METTL14 fragment (1455 bp) was cloned from pENTER [Vigene Biosciences Co. Ltd. (Jinan, China)] and inserted into the *Xba* I and *Bam*H I sites of the lentiviral vector pCDH‐EF1‐MCS‐GFP‐Puro (pLV‐con as empty vector; System Biosciences, Cat. #CD550A‐1) to generate pLV‐METTL14 (pCDH‐EF1‐METTL14‐GFP‐Puro). For METTL14 knockdown, shRNA constructs were designed in a modified pLKO.1‐puro vector [Dahong Biosciences Co. Ltd. (Guangzhou, China)], with sequences listed in Table S1.

Lentiviral packaging plasmids psPAX2 and pMD2. G, generously provided by Prof. Didier Trono (University of Geneva, Geneva, Switzerland), were used for lentivirus production. Recombinant lentiviruses, including LV‐shSCR (scrambled control shRNA), LV‐con (control), LV‐shMETTL14, and LV‐METTL14, were produced as previously described [33, 34, 35]. These lentiviruses were used to transduce CNE2, SUNE1, HONE1‐EBV, S18, HK1‐EBV, and 5‐8F cells, establishing stable cell lines expressing shSCR, vector, shMETTL14, or METTL14.

### 2.3 RNA Isolation, Reverse Transcription, and Quantitative Real‐Time PCR (qRT‐PCR)

Isolation of RNA, reverse transcription, and qRT‐PCR were carried out as outlined previously [33, 34, 35]. The primers utilized in the qRT‐PCR assay are detailed in Tables S2–S6. The endogenous control GAPDH was used for normalization, and fold changes were calculated using the relative quantification method (2^‐△△Ct^).

### 2.4 Western Blotting Assay

Western blotting was conducted as described in previous publications [33, 34, 35]. The blots were incubated with primary antibodies against GAPDH (Proteintech, Cat. No: 10494–1‐AP, rabbit, 1:10,000 dilution) or METTL14 (Sigma, Cat. No: HPA038002, rabbit, 1:1000 dilution). Hybridization signals were detected using enhanced chemiluminescence (ECL) (Millipore, Cat. No: WBKLS0500). GAPDH served as the loading control.

### 2.5 Cytokine Production Assay

Cell culture supernatants from NPC cells were pooled for 24 h and 48 h, and the concentrations in supernatants were determined by an ELISA kit [Jiangsu Meimian Industrial Co. Ltd. (Taizhou, China)] following the instructions of the manufacturer.

### 2.6 RNA Extraction, Library Preparation, and Sequencing

Total RNA was isolated using TRIzol reagent (Thermo Fisher Scientific, Cat# 15596026) according to the manufacturer's instructions. Following extraction, RNA samples were processed with DNase I (NEB, Cat# M0303L) to eliminate DNA contamination. RNA purity was assessed by measuring the A260/A280 ratio using a Nanodrop OneC spectrophotometer (Thermo Fisher Scientific Inc). RNA integrity was confirmed with the LabChip GX Touch system (Revvity). RNA concentration was quantified by a Qubit 3.0 Fluorometer and the Qubit RNA Broad Range Assay kit (Thermo Fisher Scientific, Cat# Q10210).

For library preparation, total RNA was used as input for constructing RNA sequencing libraries with the KC Digital mRNA Library Prep Kit (Cat. No. DR08502, Wuhan Seqhealth Co. Ltd., China) following the manufacturer's protocol. This kit utilizes unique molecular identifiers (UMIs) to tag preamplified cDNA molecules with 8 random bases, minimizing PCR and sequencing duplication bias. The final libraries were quantified and sequenced on the Illumina Novaseq X Plus platform using paired‐end 150 bp (PE150) sequencing.

### 2.7 Statistical Analysis

Data are expressed as mean ± SD. All statistical analyses were carried out using GraphPad 9.0 software. Comparisons of two independent groups were done using a two‐tailed Student's t test. The one‐way ANOVA helps to compare the comparisons of multiple groups. *p* values were regarded as statistically significant at *p* < 0.05, *p* < 0.01, and *p* < 0.001. NS: not significant.

## 3 Results

### 3.1 Global Analysis of Immune‐ and Inflammation‐Related Gene Expression Differences shMETT in NPC Cells with shMETTL14 Knockdown or METTL14 Overexpression

**FIGURE 1:**
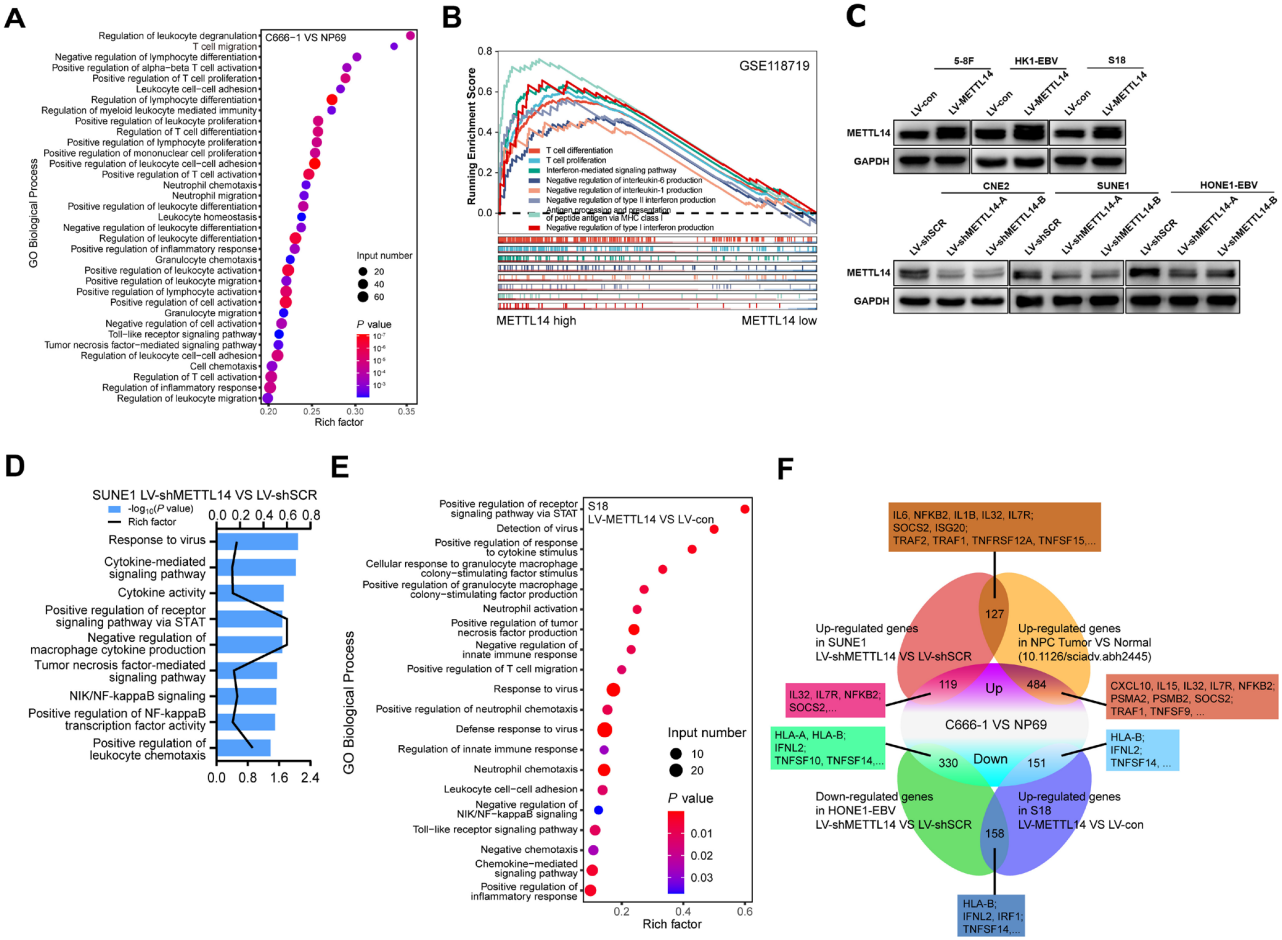
Global analysis of immune‐ and inflammation‐related gene expression differences in shMETTL14‐ or METTL14‐expressing NPC cells. (A) GO analysis of enrichment of immune‐ and inflammation‐related biological processes in both upregulated and downregulated genes in C666‐1 cells and NP69 cells. (B) The Gene Set Enrichment Analysis (GSEA) results performing functional enrichment analysis of immune‐ and inflammation‐related gene sets of some pathways in the METTL14‐high group and METTL14‐low group via the GEO (GSE118719) database. (C) METTL14 expression was analyzed by Western blot in NPC cells, which were transduced with lentivirus expressing METTL14 or shMETTL14. (D) Histogram of gene ontology (GO) term enrichment (corrected *P*‐value) analysis based on biological processes enriched in differentially expressed genes (DEGs). Gene Ontology (GO) analysis of shSCR and shMETTL14‐expressing SUNE1 cells. (E) GO analysis of up and downregulated immune response‐related genes in vector‐expressing (LV‐con) and METTL14‐expressing (LV‐METTL14) S18 cells. (F) Venn diagram for the overlap of DEGs based on analysis of RNA‐seq data from C666‐1, NPC tumors, METTL14‐expressing S18 cells, and shMETTL14‐expressing NPC cells (SUNE1 and HONE1‐EBV).

To further examine the expression profiles of immune‐ and inflammation‐related genes in NPC cells, we conducted RNA‐seq in C666‐1 (EBV‐positive NPC) and NP69 (normal control) cells. In total, there were 6487 differentially expressed genes (DEGs) among the two groups using differential expression analysis, which were annotated by Gene Ontology (GO) classification. GO enrichment analysis indicated that these DEGs were markedly overrepresented in immune‐ and inflammation‐related pathways (Figure 1A), including TNF, IFN, IL, and MHC I signaling pathways. Likewise, the DEGs between S18 and NP69 cells, as well as between NPC tissues and normal tissues, were also enriched in these aforementioned pathways (Figures S1A and S1B). Functional enrichment analysis performed based on the GEO (GSE118719, GSE102349) database indicated that immune‐ and inflammation‐related pathways showed substantial enrichment in the METTL14 high‐expression group (Figures 1B and Figure S1C). In addition, the overlap analysis of 2434 upregulated genes in C666‐1 cells and NPC tissues revealed that 484 of them are overlapped, as well as some immune‐ and inflammation‐related genes including CXCL10, IL15, IL32, IL7R, NFKB2, PSMA2, PSMB2, SOCS2, TRAF1, and TNFSF9 (Figure 1F).

To investigate whether METTL14 drives immune‐ and inflammation‐related gene expression, we constructed stable METTL14‐overexpressing and METTL14‐knockdown NPC cell lines. Consistently, qRT‐PCR and Western blot assays demonstrated that, in the METTL14‐overexpressing NPC cells, METTL14 expression was significantly upregulated, and in the METTL14‐knockdown NPC cells, METTL14 expression was downregulated (Figure S1D and Figure 1C). RNA‐seq analysis demonstrated that the DEGs upon METTL14 knockdown were significantly enriched in TNF, IFN, IL, and MHC I‐associated pathways (Figure 1D) and Figure S1E. GSEA analysis also validated that these DEGs correlated with immune or inflammatory response Figure S1F. Likewise, a similar immune or inflammatory response was significantly enriched in DEGs of METTL14‐overexpressed S18 cells (Figure 1E). Overlapping analysis of downregulated genes in METTL14‐knockdown HONE1‐EBV cells and genes with elevated expression in METTL14‐overexpressing S18 cells was performed to further explore whether METTL14 regulated NPC‐related immune‐ and inflammation‐related genes, and 158 overlapping genes were identified (Figure 1F), including immune‐ and inflammation‐related genes such as HLA‐B, IFNL2, IRF1, and TNFSF14 (Figure 1F). Moreover, overlapping between upregulated genes in METTL14‐knockdown SUNE1 cells and C666‐1 cells revealed several immune‐ and inflammation‐related upregulated genes, such as IL32, IL7R, NFKB2, and SOCS2 (Figure 1F). The above findings revealed that METTL14 might drive immune action and inflammation in NPC, especially through TNF, IFN, IL, and MHC I‐related genes in shMETTL14‐ or METTL14‐expressing cells.

### 3.2 METTL14 Altered the Expression of TNF‐Related Genes in NPC Cells

**FIGURE 2:**
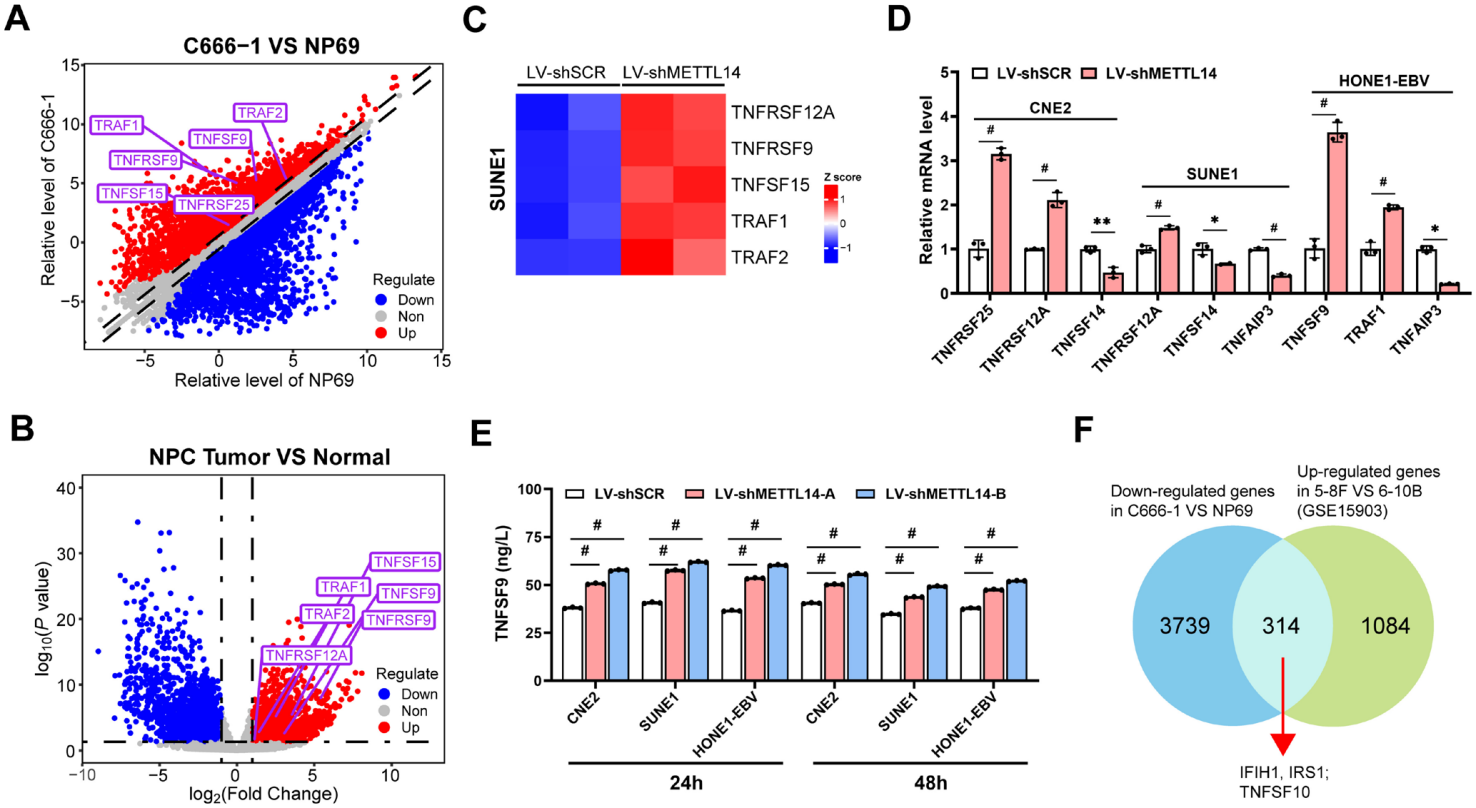
METTL14 altered the expression of TNF‐related genes in NPC cells. (A) Scatter plots depicting the impact of TNF‐related gene expression on C666‐1 cells and NP69 cells. Based on the treatment effect, repressed genes are represented in blue, activated genes in red, and those with nonsignificant (1.5‐fold) changes in expression are shown in gray. (B) Volcano plot of TNF‐related genes differentially expressed in NPC tumors and normal tissues. (C) Heatmap was used for visualization of the differentially expressed TNF‐related genes between shSCR‐ and shMETTL14‐expressing SUNE1 cells, with genes upregulated in red and downregulated in blue. (D) qRT‐PCR measurement of TNF‐regulated gene expression in CNE2, SUNE1 and HONE1‐EBV shMETTL14‐expressing cells. (E) Supernatants from shMETTL14‐expressing NPC cells (CNE2, SUNE1, and HONE1‐EBV) were collected at 24 h and 48 h, and TNFSF9 levels were measured. (F) Overlap of differentially expressed genes (DEGs) in paired C666‐1 cells and 5‐8F cells, shown as a Venn diagram.

Consequently, to better understand whether METTL14 modulates TNF‐related gene expression, we analyzed DEGs of TNF‐related genes between C666‐1 and NP69 cells. The most prominently downregulated and elevated TNF‐related genes are shown in the Volcano plots (Figure 2A), including TNFSF15, TNFRSF9, TNFSF9, and TNFRSF25, which were dramatically upregulated in C666‐1 cells. Consistently, these TNF‐related genes were also significantly upregulated in NPC tumor tissues (Figure 2B). RNA‐seq analyses showed that knockdown of METTL14 significantly increased expression of a number of TNF‐related genes such as TNFRSF12A, TNFRSF9, TNFSF15, TRAF1, and TRAF2 (Figure 2C). In contrast, METTL14 overexpression significantly decreased TNFRSF12A, TNFSF9, and TRAF2 expression, but increased TNFAIP3, TNFSF10, and TNFSF14 (Figure S2A). qRT‐PCR analysis consistently showed that METTL14‐overexpression and METTL14‐knockdown NPC cells generally displayed upregulation of TNF‐related gene expression (Figure S2B, C, and 2D). ELISA analysis revealed that METTL14‐knockdown NPC cells significantly increased TNFSF9 production compared to controls, with notable elevations observed at both 24 h and 48 h (Figure 2E). To refine further, we performed an analysis that intersected significantly downregulated genes in C666‐1 cells as compared to NP69 cells with significantly upregulated genes in 5‐8F (highly metastatic NPC cells) versus 6‐10B (low metastatic NPC cells) within the GSE15903 dataset and identified 314 overlapping genes (among others IFIH1, IRS1, TNFSF10) (Figure 2F). Overall, our findings demonstrate that METTL14 knockdown in NPC cells upregulates TNF‐associated genes, highlighting its critical role in regulating pathogenesis and immune responses in NPC.

### 3.3 Induction of IFN‐Regulated Gene Expression by RNAi‐Mediated METTL14 Silencing in NPC Cells

**FIGURE 3:**
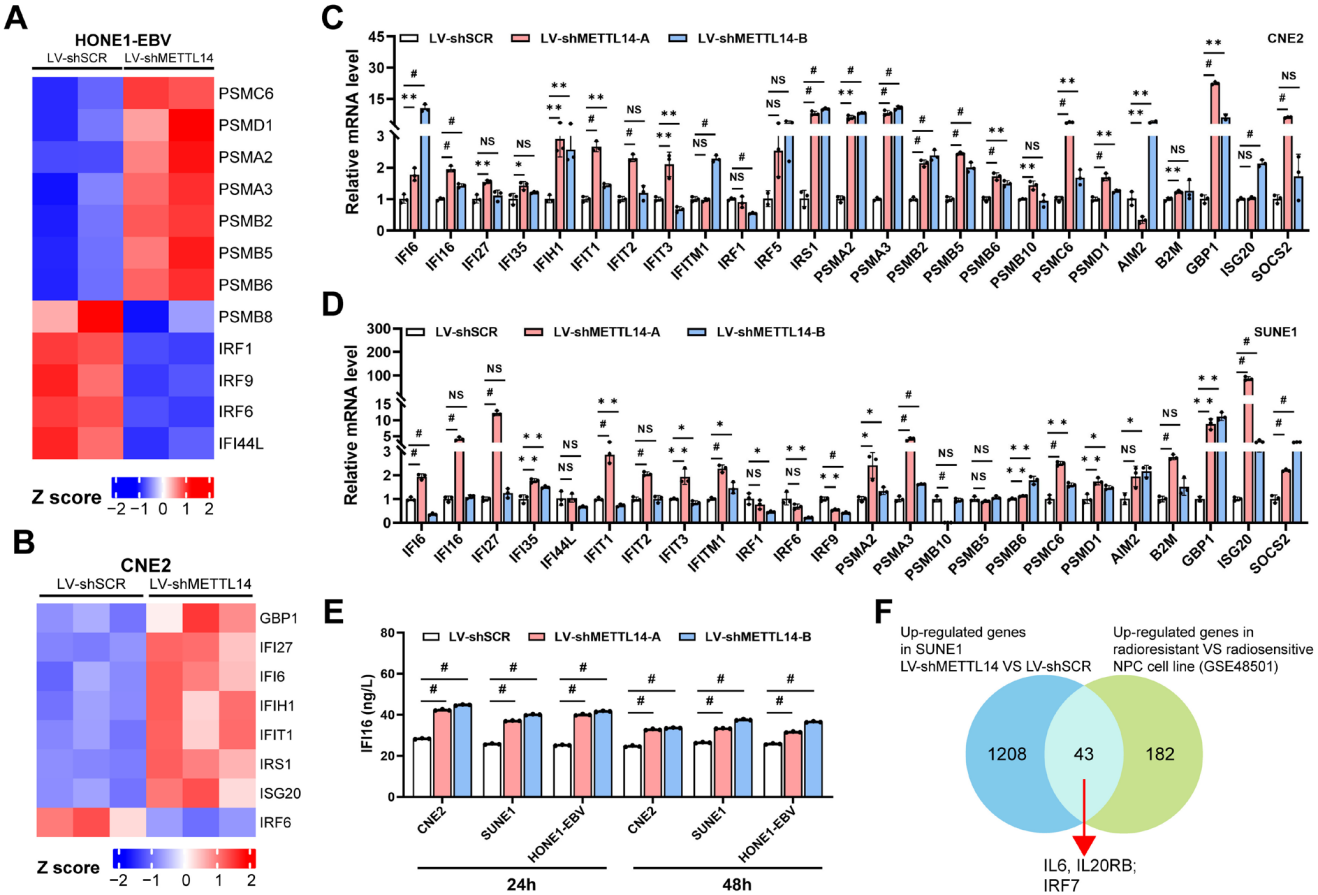
Induction of IFN‐regulated gene expression by RNAi‐mediated METTL14 silencing in NPC cells. (A‐B) Heatmap showing the differential expression of IFN‐related genes of shSCR‐ vs. shMETTL14‐expressing HONE1‐EBV (A) and CNE2 (B) cells. (C‐D) IFN‐induced gene expression analyzed by qRT‐PCR in CNE2 (C) and SUNE1 (D) cells expressing shMETTL14. (E) Supernatants from shMETTL14‐expressing NPC cells were collected at 24 h and 48 h, and IFI16 levels were measured. (F) Overlap of differentially expressed genes (DEGs) in paired shMETTL14 expressing NPC cells and radiosensitive NPC cells, shown as a Venn diagram.

Figure 1 indicates that METTL14 may regulate immune‐ and inflammation‐related gene expression, such as genes associated with IFN, in NPC cells. To validate whether METTL14 affects genes regulated by IFN, we performed RNA‐seq analysis in C666‐1 cells and NP69 cells, respectively. Using a scatter plot (Figure S3A), we clearly showed that many IFN‐regulated genes were significantly upregulated, including PSMA2, PSMB2, PSMB5, PSMC6, IRF5, and SOCS2, while IFI44L and PSMB10 were significantly downregulated. GO enrichment analysis likewise demonstrated that these DEGs are involved in the cellular response to type I interferon as well as the interferon‐γ pathways (Figure S3B). Moreover, IFN‐regulated genes were substantially increased in NPC tumor tissues in comparison to normal tissues (Figure S3C). With the aim of investigating the function of METTL14 on IFN‐regulated genes, we undertook RNA‐seq analysis of NPC cells with METTL14 overexpression or knockdown. Still, elevated METTL14 expression in S18 cells led to a pronounced rise in multiple IFN‐regulated genes (PSMB8, IRF1, IRF6, IRF9), in addition to a significant downregulation of IFI27, IRS, PSMB2, PSMB5, PSMB6, and SOCS2 (Figure S4A). By contrast, METTL14 knockdown in HONE1‐EBV cells significantly elevated the expression of PSMC6, PSMD1, PSMA2, PSMA3, PSMB2, PSMB5, and PSMB6, and significantly decreased the expression of PSMB8, IRF1, IRF9, IRF6, and IFI44L (Figure 3A). Comparable findings were obtained in CNE2 and SUNE1 cells with METTL14 knockdown (Figure 3B and S4B–D). Subsequent analysis using the overlapping genes intersected between the upregulated genes in SUNE1 LV‐shMETTL14 compared with LV‐shSCR and the upregulated genes in radioresistant compared with radiosensitive NPC cell lines (GSE48501) revealed that 43 overlapping genes, including IL6, IL20RB, and IRF7, were also significantly upregulated (Figure 3F). qRT‐PCR analysis revealed a broad IFN‐activated gene upregulation in METTL14‐depleted NPC cells (Figure 3C,D and S4E), though IFI44L, IRF1, and IFNL2 expression was paradoxically downregulated in both HONE1‐EBV and HONE1 knockdown models (Figure S4E,F). IFI16 production was significantly upregulated in METTL14‐knockdown NPC cells, as shown by ELISA analysis, with elevated levels at both 24 h and 48 h (Figure 3E). To sum up, the depletion of METTL14 greatly enhances the regulation of IFN‐induced genes in NPC cells.

### 3.4 Loss of METTL14 Expression Upregulated the Expression of IL‐Related Genes in NPC Cells

**FIGURE 4:**
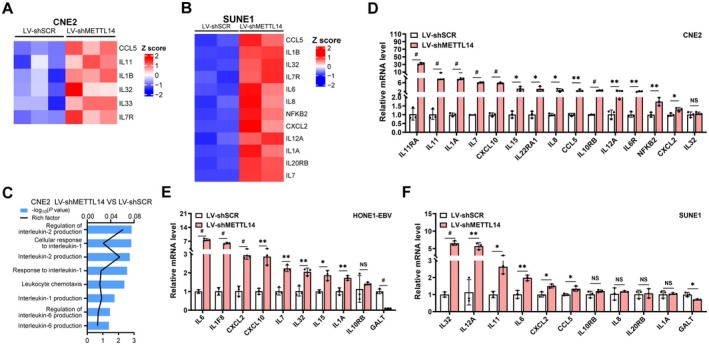
Loss of METTL14 expression upregulated the expression of IL‐related genes in NPC cells. (A‐B) Heatmap of IL‐related genes in CNE2 (A) and SUNE1 (B) cells expressing either shSCR or shMETTL14. (C) The histogram of GO term enrichment analysis of IL‐related genes in shSCR‐ and shMETTL14‐expressing CNE2 cells. (D‐F) qRT‐PCR for IL‐regulated gene expression in CNE2 (D), HONE1‐EBV (E) and SUNE1 (F) cells expressing shMETTL14.

Genes commonly associated with IL‐associated pathways play pivotal roles in regulating immune responses and inflammatory processes. To verify whether METTL14 regulated IL‐related genes, we performed RNA‐seq analysis in C666‐1 and NP69 cells. Accordingly, the volcano plot detected remarkable upregulation of various IL genes, such as IL10RB, IL17D, IL7R, IL7, CXCL10, IL32, IL15, and NFKB2 (Figure S5A). Comparative analysis revealed significant upregulation of these genes in NPC tumor tissues in contrast to normal tissues (Figure S5B). Subsequent GO analysis, performed using the enrich GO package, further substantiated the functional relevance of these DEGs, demonstrating their significant enrichment in IL‐associated pathways (Figure S5C,D). GSEA analysis also showed the significant enrichment of interleukin‐1 responsive genes in NPC tumor tissues (Figure S5E,F). Based on RNA‐seq analysis, we observed that METTL14 overexpression was reduced several IL‐associated genes, including IL11RA, IL20RB, IL32, and IL7 (Figure S6A). GO enrichment and GSEA analyses suggested that these DEGs were closely related to IL‐associated pathways (Figure S6B,C). In contrast, IL‐related genes were upregulated after METTL14 knockdown (CCL5, IL1B, IL32, IL7R, IL6, IL7, IL8, NFKB2, CXCL2, IL12A, IL1A, and IL20RB) (Figures 4A,B and S6D). Pathway enrichment for GO analysis and GSEA consistently revealed involvement of IL‐associated pathways (Figures 4C and S6E,F). qRT‐PCR revealed that tens of IL‐related gene expression levels have increased overall in NPC cells upon METTL14 knockdown (Figures 4D–F and S6G), which is opposite to the observation in the other direction. In summary, METTL14 depletion drastically elevates the expression of the IL‐related genes in NPC cells, further highlighting METTL14's crucial function in the regulation of IL‐associated pathways in NPC.

### 3.5 METTL14 Knockdown Led to Elevated Production of IL‐6, IL‐7, and CXCL10 in NPC Cells

**FIGURE 5:**
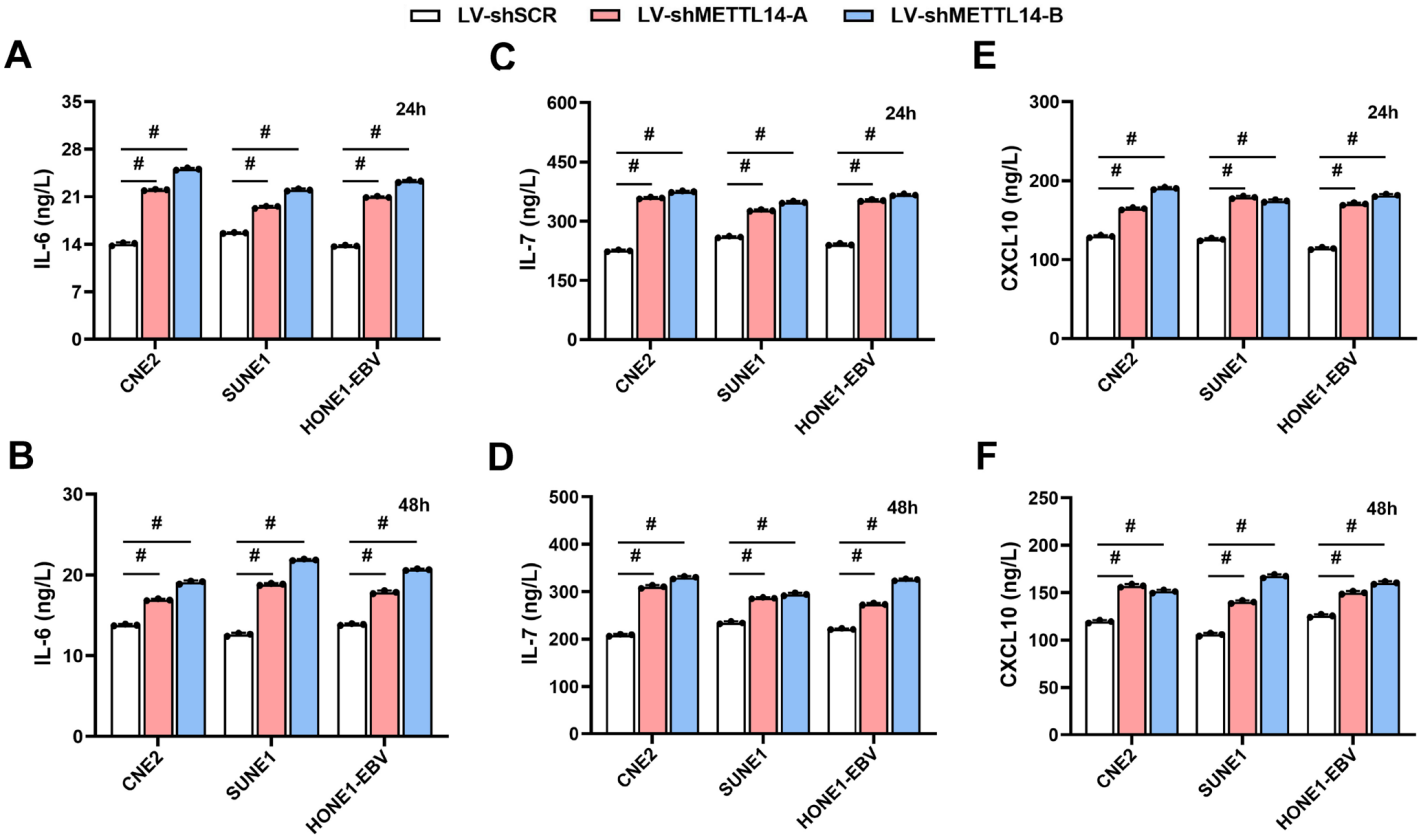
METTL14 knockdown led to elevated production of IL‐6, IL‐7, and CXCL10 in NPC cells. Supernatants of NPC cells expressing shMETTL14 were collected for 24 h and 48 h, then assayed for IL6, IL7, and CXCL10 levels in CNE2 (A‐B), SUNE1 (C‐D), and HONE1‐EBV (E‐F) cells.

Since METTL14 knockdown induces expression of IL‐6, IL‐7, and CXCL10 in NPC cells (Figure 4D–F), we further examined whether exogenous depletion of METTL14 could modulate NPC cell‐derived production of these cytokines. Remarkably, METTL14 knockdown in CNE2, SUNE1, and HONE1‐EBV cells triggered a marked increase in IL‐6, IL‐7, and CXCL10 production compared to their respective controls, with significant elevation observed at both 24 h (Figure 5A,C,E) and 48 h (Figure 5B,D,F). These results suggest that METTL14 promotes IL‐6, IL‐7, and CXCL10 secretion in NPC cells.

### 3.6 METTL14 Adjusted the Regulation of MHC Class I Genes in NPC Cells

**FIGURE 6:**
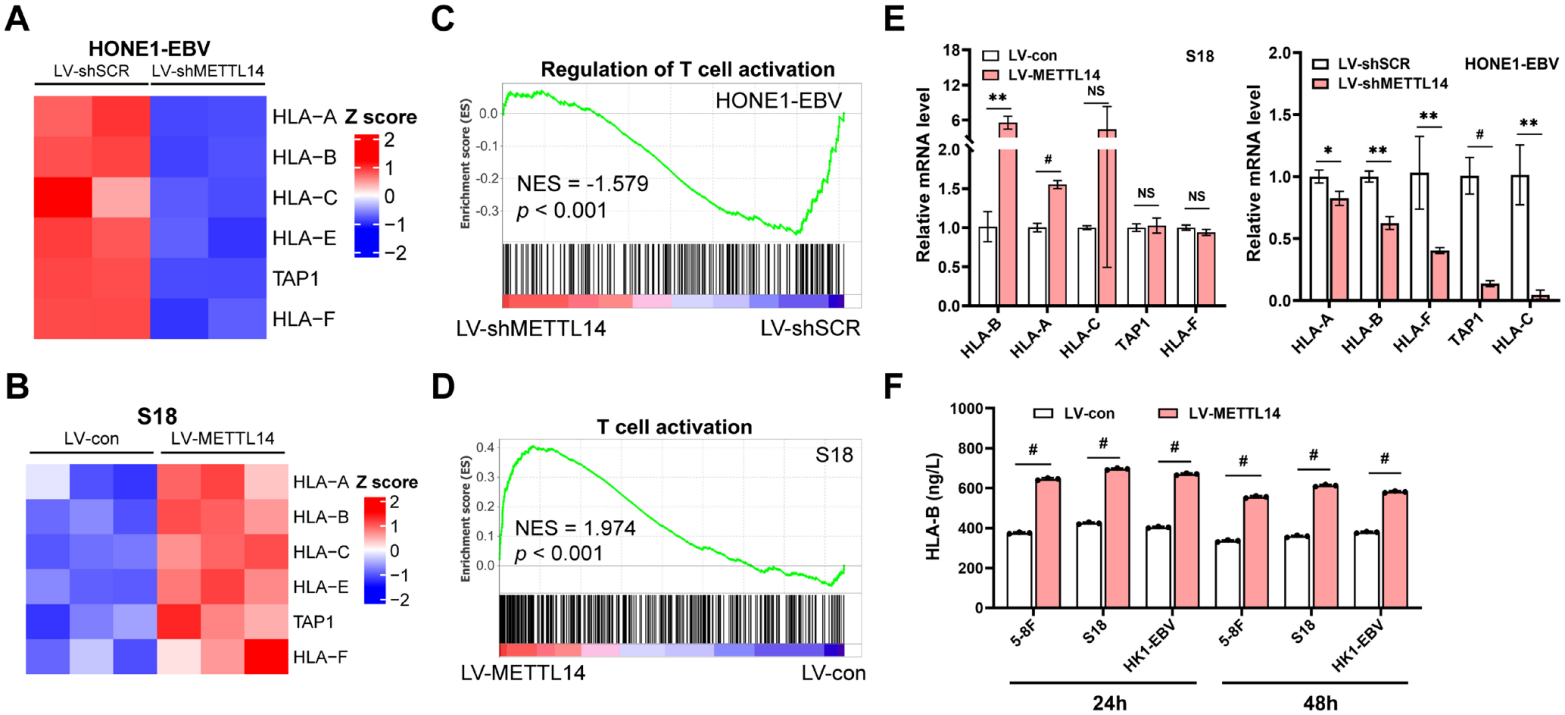
METTL14 adjusted the regulation of MHC class I genes in NPC cells. (A) Heatmap plots of differentially expressed MHC class I genes between control and METTL14‐expressing S18 cells. (B) Heatmap showing the differential expression of MHC class I‐encoding genes in HONE1‐EBV cells expressing shSCR and shMETTL14. (C) GSEA plot of enrichment of MHC class I genes in control and METTL14‐expressing S18 cells. (D) GSEA plot of enrichment of MHC class I genes in shSCR‐ and shMETTL14‐expressing HONE1‐EBV cells. (E) qRT‐PCR analysis of MHC class I genes in METTL14‐overexpressing (left) and shMETTL14‐expressing (right) NPC cells. (F) Supernatants from shMETTL14‐expressing NPC cells were collected at 24 h and 48 h, and HLA‐B levels were measured.

Indeed, although MHC class I complexes are broadly displayed on all cells with a nucleus in a constitutive manner, evidence is accumulating that MHC class I antigens are downregulated in tumor cells to evade immune surveillance. Analysis of volcano plots showed that MHC class I gene expression of HLA‐A and ‐B was substantially increased in C666‐1 cells than in NP69 cells (Figure S7A). GO enrichment analysis of C666‐1 vs. NP69 revealed that the DEGs were intimately linked to T cell activation, T cell differentiation (Figure S7B). Likewise, GO enrichment analysis of DEGs between the NPC tumor tissues and normal tissues also revealed significant enrichment in T cell and B cell activation and differentiation‐related pathways (Figure S7C). To explore the influence of METTL14 on the expression of MHC class I genes in NPC cells, we first conducted heatmap analysis to show that METTL14 overexpression markedly upregulated MHC class I genes (HLA‐A, B, C, E, F, and TAP1) (Figure 6A), whereas METTL14 depletion led to substantial downregulation of the above genes (Figure 6B). GSEA analysis also showed that METTL14 expression changes were significantly linked with genes in T cell activation (Figures 6C,D and S7D‐F). MHC class I genes such as HLA‐B, ‐C, ‐E, and ‐A were upregulated in METTL14‐overexpressing S18 and HK1 cells, as revealed by qRT‐PCR (Figure 6E and S7G). Alternatively, HONE‐EBV and HONE1 cells with METTL14 knockdown had the opposite downregulation of genes (Figure 6E and S7H). In METTL14‐overexpression NPC cells, ELISA analysis demonstrated a significant upregulation of HLA‐B production, with elevated levels at both 24 h and 48 h (Figure 6F). Interestingly, METTL14 regulates the expression of MHC class I genes in NPC cells.

## 4 Discussion

Accumulating evidence supports the idea that m^6^A RNA methylation, mediated by methyltransferases, is especially fundamental to immune and inflammation‐related diseases, including rheumatoid arthritis [36, 37], psoriasis [23], systemic lupus erythematosus (SLE) [38], chronic kidney disease [39], asthma [40], acute lung injury [41], atherosclerosis [21, 42], Crohn's disease [27], ulcerative colitis [43], and allergic reactions [40]. For example, METTL14 promotes the activation of fibroblast‐like synoviocytes (FLS) through the LASP1‐SRC‐AKT axis in rheumatoid arthritis [36]. Additionally, by inducing apoptosis of RA‐FLS, METTL3 contributes to rheumatoid arthritis [37]. Through its interaction with METTL14, UCA1 activates both HIF‐1α and NF‐κB signaling pathways, consequently promoting keratinocyte‐mediated inflammatory responses and contributing to psoriasis pathogenesis [23]. METTL3 promotes IRF4 expression in an m^6^A‐regulated manner, leading to plasma cell accumulation and subsequent SLE kidney injury [38]. Furthermore, METTL3‐mediated m^6^A modification of EVL attenuates the progression of kidney fibrosis [39]. METTL3 suppresses M2 macrophage activation and subsequent asthma development through the PI3K‐AKT and JAK‐STAT6 signaling pathways [40]. METTL14 stabilizes NLRP3 expression via IGF2BP2‐mediated stabilization, contributing to acute lung injury [41]. METTL14‐mediated m^6^A modification regulates FOXO1 expression, while METTL3‐dependent m^6^A methylation modulates IGF2BP1 activity, collectively promoting angiogenesis and exacerbating atherosclerotic progression [21] [42],. METTL14 regulates the NF‐κB pathway to prevent colon epithelial cell death [27]. METTL14 also protects against colonic inflammation by regulating the DHRS4‐AS1/miR‐206/A3AR pathway [43]. Finally, METTL3 modulates allergic asthma by regulating M2 macrophage promotion mediated by the PI3K‐AKT and JAK‐STAT6 signaling pathways [40].

Chronic inflammation is considered to contribute significantly to cancer initiation and progression, serving as a significant risk factor by creating a microenvironment conducive to tumor initiation, progression, and metastasis [44]. Chronic inflammation serves as a crucial driver in tumor development, impacting multiple stages of tumor progression through diverse mechanisms, including DNA damage induction, angiogenesis promotion, enhancement of tumor invasion, metastasis, metabolic reprogramming, and remodeling of the tumor microenvironment [45, 46, 47]. Increasing evidence indicates that m^6^A methyltransferases mediate inflammatory responses that drive tumor progression. A majority of tumors mobilize tumor‐associated macrophages (TAMs) to foster a supportive microenvironment for tumor expansion. METTL3 has been shown to facilitate tumor progression by recruiting TAMs and inhibiting M1 macrophage polarization [48, 49]. Additionally, METTL3 knockdown in thyroid cancer cells enhances the secretion of pro‐inflammatory cytokines, particularly IL‐8, thereby promoting neutrophil infiltration [50]. The acidic tumor microenvironment suppresses T‐cell recruitment and impairs anti‐tumor immunity by downregulating the METTL3‐m^6^A‐integrin *β*1 signaling pathway [51]. Moreover, m^6^A‐modified lncRNA TUG1 negatively regulates anti‐tumor immunity by inhibiting CD8+ T‐cell activation in HCC [52]. In contrast, IL‐37 upregulates METTL14 expression in lung cancer, thereby enhancing CD8+ T‐cell infiltration [53]. Furthermore, METTL14 knockdown has been associated with increased tumor immunogenicity, as evidenced by enhanced CD8+ T‐cell infiltration and elevated secretion of IFN‐γ and CXCL9/10 within the tumor microenvironment [30]. Moreover, in our study, METTL14 knockdown significantly upregulated TNF‐related genes (e.g., TNFSF9 and TNFRSF25), IFN‐associated genes (e.g., IFI6, IFI16, IFIH1, and IRF5), and IL‐related genes (e.g., IL6, IL7, and CXCL10). Conversely, METTL14 overexpression enhanced MHC class I expression, particularly HLA‐A, B, C, and –E. Collectively, our results provide strong evidence that METTL14 serves as a key regulator in linking inflammation and cancer.

IL‐6, a multifunctional cytokine, is instrumental in tumorigenesis by driving cell growth, survival, metastasis, and drug resistance through the activation of the JAK/STAT3 signaling pathway [54, 55, 56, 57]. IL‐6 is commonly associated with chronic inflammatory responses in the tumor microenvironment, where it regulates immune cell function and promotes immune evasion of tumors [58, 59, 60]. Furthermore, IL‐6 contributes to tumor progression by promoting angiogenesis [61, 62], tumor infiltration [63], and immune suppression [64, 65], creating a favorable environment for tumor growth. Elevated expression of IL‐6 and activation of the JAK2/STAT3 pathway are tightly correlated with the progression of malignancies, including HCC [65], colon cancer [66], bc [ 67], NSCLC [56], and NPC [68]. Elevated IL‐6 production and activation of the JAK2/STAT3 pathway are associated with poor prognosis and reduced survival in NPC patients [68]. FKBP3 suppresses NPC cell proliferation, invasion, and migration via the NF‐κB/IL‐6 pathway [69], while lncRNA DANCR promotes NPC progression through IL‐6/JAK1/STAT3 upregulation [70]. Exosome‐mediated IL‐6 secretion by NPC cells facilitates tumor progression [68]. Additionally, a positive correlation exists between EBV DNA load, IL‐6, and VEGF expression, with higher plasma EBV DNA and elevated IL‐6/VEGF levels serving as potential indicators of NPC recurrence and disease progression [71]. These findings strongly indicate that METTL14‐mediated modulation of IL‐6 expression may be crucial for NPC progression, necessitating further investigation.

IFI16 plays a crucial role in immune surveillance and tumor regulation through its inflammasome‐associated “sensing‐response” mechanism [72]. It mediates noncanonical STING activation, inducing NF‐κB signaling in HER2+ bc [73, 74], while its knockout suppresses ESCC proliferation, IL‐1α secretion, and tumor progression [75, 76]. Additionally, IFI16 enhances type I IFN‐mediated antitumor effects in triple‐negative BC by impairing DNA repair [77]. This study identifies METTL14 silencing in NPC cells that markedly upregulates IFN‐associated genes, including IL16, IFI16, IFIH1, IRF5, IRS1, PAMA2, PSMA3, and SOCS2, representing a novel regulatory mechanism distinct from other known physiological or pathological conditions. Moreover, extracellular IFI16 promotes TLR4‐mediated inflammation via LPS binding [78], sustains STING expression to activate IFN‐γ signaling in melanoma [79], and exerts antitumor effects by inducing apoptosis, inhibiting neovascularization, and enhancing macrophage recruitment [80]. IFI16 also mediates Nutlin‐3‐induced chromatin redistribution and p53 activation in HCC [81]. In conclusion, the findings of this study, supported by published research, suggest with confidence that METTL14's regulation of IFN‐induced gene expression likely plays a pivotal role in the pathogenesis of NPC, which requires further exploration.

MHC class I molecules, essential components of the immune system, present endogenous antigens, including tumor antigens, to cytotoxic T cells (CD8+ T cells), triggering immune responses. These molecules consist of classical MHC class I molecules (HLA‐A, B, and C) and nonclassical MHC class I molecules (HLA‐E, F, G, H, and J). HLA‐B molecules are crucial for presenting intracellular neoplasm‐specific antigens for recognition by CD8+ T cells, activating cytotoxic T lymphocyte responses. This process enables the immune system to discern and eliminate neoplastic cells. In pancreatic cancer, HLA‐B modulates ITGB1 expression, influencing migration and transmembrane signaling [82], while its altered expression impairs tumor antigen recognition in cervical cancer [83] and affects immune checkpoint inhibitor efficacy in gastrointestinal malignancies [84]. Additionally, HLA‐B plays a role in inhibiting colorectal cancer growth [85], with MSI cases exhibiting a C6/C7 tandem repeat in exon 4 [86]. This study reveals that METTL14 overexpression in NPC cells upregulates MHC class I genes (HLA‐A, B, C, −E), whereas METTL14 silencing broadly downregulates MHC class I expression, including HLA‐A, B, C, F, −G, and TAP1. This study offers fresh perspectives on the possible function of METTL14 in tumor immune evasion through the suppression of cell surface HLA class I expression, although further characterization is needed.

While our in vitro models provide valuable mechanistic insights into the role of METTL14 in the regulation of immune‐ and inflammation‐related genes, they inherently lack the complexity of the tumor microenvironment (TME) and the systemic interactions that occur in vivo. Key factors such as immune cell infiltration [42], stromal interactions [87], angiogenesis [88], and metabolic adaptations [89] within the TME may significantly modulate METTL14‐dependent phenotypes. Consequently, our findings, derived primarily from monoculture systems, may not fully recapitulate the pathophysiological context of human tumors. Future validation using in vivo models, such as patient‐derived xenografts or genetically engineered mouse models, alongside correlative analyses of METTL14 expression/activity in clinical NPC specimens, will be essential to confirm the translational relevance of these observations.

Although this study provides comprehensive transcriptomic profiling of both nasopharyngeal carcinoma (NPC) cells and tissues, we acknowledge that the presence of Epstein–Barr virus (EBV) in C666‐1 cells may influence the observed differential gene expression patterns. Current evidence demonstrates that EBV can extensively modulate host cell gene expression through its latent gene products [90, 91] and noncoding RNAs [92, 93]. Nevertheless, this potential confounding factor precisely highlights the significance of our study, as we specifically focus on elucidating EBV‐mediated regulation of immune‐ and inflammation‐related genes via METTL14 in NPC. The identified immune and inflammatory signatures, particularly the upregulation of cytokines including CXCL10, IL‐6, TNFSF9, and HLA‐B, may reflect both intrinsic tumor biology and EBV‐induced immunomodulation, all of which warrant further investigation into EBV–host interactions in NPC pathogenesis.

In summary, our findings demonstrate that METTL14 regulates immune‐ and inflammation‐related genes, including TNF, IFN, IL, and MHC class I, in NPC. Nevertheless, the downstream effects of METTL14‐mediated modulation of immune or inflammation‐related genes in NPC cells warrant further exploration. Overall, we propose that METTL14 could be involved in cancer progression by modulating the expression of these immune and inflammation‐associated genes.

## Author Contributions

**Zhihao Zhou:** conceptualization (lead), data curation (lead), investigation (lead), writing – original draft (equal), writing – review and editing (equal). **Jing Wang:** conceptualization (lead), data curation (lead), investigation (lead), writing – original draft (equal), writing – review and editing (equal). **Lingjun Shen:** conceptualization (equal), funding acquisition (lead), investigation (equal). **Liuxin Han:** investigation (equal), resources (equal). **Qiwen Li:** conceptualization (lead), data curation (lead), investigation (lead). **Aibing Wu:** conceptualization (equal), data curation (equal), investigation (equal), resources (lead), writing – original draft (lead), writing – review and editing (lead). **Jing Li:** funding acquisition (equal), resources (equal). **Zuming Liang:** investigation (equal). **Litong Zhu:** investigation (equal). **Danhua He:** investigation (equal). **Ying Zhou:** investigation (equal), resources (equal). **Shihao Huang:** investigation (equal). **Zhanlin Zhao:** investigation (equal). **Jinge Cong:** investigation (equal). **Zhitao Peng:** investigation (equal). **Ping Zhao:** investigation (equal). **Shuna Ye:** investigation (equal). **Binyi Bai:** investigation (equal). **Xuanjia Hong:** investigation (equal). **Guanqi Dai:** investigation (equal). **Ye Lei:** investigation (equal). **Wentao Zhao:** funding acquisition (lead), resources (equal). **Junshuang Jia:** investigation (equal). **Xiaolin Lin:** investigation (equal). **Dong Xiao:** conceptualization (lead), data curation (lead), funding acquisition (lead), resources (lead), supervision (lead), visualization (lead), writing – original draft (lead), writing – review and editing (lead). **Yuqin Zhang:** conceptualization (lead), data curation (lead), funding acquisition (lead), supervision (lead), writing – original draft (lead), writing – review and editing (lead). **Taoyan Lin:** conceptualization (lead), data curation (lead), funding acquisition (lead), supervision (lead), writing – original draft (equal), writing – review and editing (lead).

## Ethics Statement

The authors have nothing to report.

## Conflicts of Interest

The authors declare no conflicts of interest.

## Supporting information


**Data S1:** cam471371‐sup‐0001‐TableS1‐S6‐FigureS1‐S7.docx.

## Data Availability

Data are contained within the article or Supporting Information.

## References

[1] Y. P. Chen , A. T. C. Chan , Q. T. Le , P. Blanchard , Y. Sun , and J. Ma , “Nasopharyngeal Carcinoma,” Lancet 394 (2019): 64–80, 10.1016/S0140-6736(19)30956-0. 31178151

[2] M. L. K. Chua , J. T. S. Wee , E. P. Hui , and A. T. C. Chan , “Nasopharyngeal Carcinoma,” Lancet 387 (2016): 1012–1024, 10.1016/S0140-6736(15)00055-0. 26321262

[3] L. Chen , Y. Zhang , S. Z. Lai , et al., “10‐Year Results of Therapeutic Ratio by Intensity‐Modulated Radiotherapy Versus Two‐Dimensional Radiotherapy in Patients With Nasopharyngeal Carcinoma,” Oncologist 24 (2019): e38–e45, 10.1634/theoncologist.2017-0577. 30082487 PMC6324627

[4] X. R. Tang , Y. Q. Li , S. B. Liang , et al., “Development and Validation of a Gene Expression‐Based Signature to Predict Distant Metastasis in Locoregionally Advanced Nasopharyngeal Carcinoma: A Retrospective, Multicentre, Cohort Study,” Lancet Oncology 19 (2018): 382–393, 10.1016/S1470-2045(18)30080-9. 29428165

[5] Y. An and H. Duan , “The Role of m6A RNA Methylation in Cancer Metabolism,” Molecular Cancer 21 (2022): 14, 10.1186/s12943-022-01500-4. 35022030 PMC8753874

[6] L. He , H. Li , A. Wu , Y. Peng , G. Shu , and G. Yin , “Functions of N6‐Methyladenosine and Its Role in Cancer,” Molecular Cancer 18 (2019): 176, 10.1186/s12943-019-1109-9. 31801551 PMC6892141

[7] C. Zeng , W. Huang , Y. Li , and H. Weng , “Roles of METTL3 in Cancer: Mechanisms and Therapeutic Targeting,” Journal of Hematology and Oncology 13 (2020): 117, 10.1186/s13045-020-00951-w. 32854717 PMC7457244

[8] Q. Guan , H. Lin , L. Miao , et al., “Functions, Mechanisms, and Therapeutic Implications of METTL14 in Human Cancer,” Journal of Hematology and Oncology 15 (2022): 13, 10.1186/s13045-022-01231-5. 35115038 PMC8812173

[9] E. Sendinc and Y. Shi , “RNA m6A Methylation Across the Transcriptome,” Molecular Cell 83 (2023): 428–441, 10.1016/j.molcel.2023.01.006. 36736310

[10] X. Y. Chen , J. Zhang , and J. S. Zhu , “The Role of m(6)A RNA Methylation in Human Cancer,” Molecular Cancer 18 (2019): 103, 10.1186/s12943-019-1033-z. 31142332 PMC6540575

[11] Y. Yang , J. Cai , X. Yang , et al., “Dysregulated m6A Modification Promotes Lipogenesis and Development of Non‐Alcoholic Fatty Liver Disease and Hepatocellular Carcinoma,” Molecular Therapy 30 (2022): 2342–2353, 10.1016/j.ymthe.2022.02.021. 35192934 PMC9171149

[12] X. Bai , J. Liu , S. Zhou , L. Wu , X. Feng , and P. Zhang , “METTL14 Suppresses the Expression of YAP1 and the Stemness of Triple‐Negative Breast Cancer,” Journal of Experimental & Clinical Cancer Research 43 (2024): 307, 10.1186/s13046-024-03225-2. 39563370 PMC11577812

[13] M. Wang , J. Liu , Y. Zhao , et al., “Upregulation of METTL14 Mediates the Elevation of PERP mRNA N(6) Adenosine Methylation Promoting the Growth and Metastasis of Pancreatic Cancer,” Molecular Cancer 19 (2020): 130, 10.1186/s12943-020-01249-8. 32843065 PMC7446161

[14] Y. Chen , S. Bian , J. Zhang , et al., “HSV‐1‐Induced N6‐Methyladenosine Reprogramming via ICP0‐Mediated Suppression of METTL14 Potentiates Oncolytic Activity in Glioma,” Cell Reports 43 (2024): 114756, 10.1016/j.celrep.2024.114756. 39325621

[15] H. Weng , H. Huang , H. Wu , et al., “METTL14 Inhibits Hematopoietic Stem/Progenitor Differentiation and Promotes Leukemogenesis via mRNA m(6)A Modification,” Cell Stem Cell 22 (2018): 191–205, 10.1016/j.stem.2017.11.016. 29290617 PMC5860916

[16] X. Ji , X. Wan , H. Sun , et al., “METTL14 Enhances the m6A Modification Level of lncRNA MSTRG.292666.16 to Promote the Progression of Non‐Small Cell Lung Cancer,” Cancer Cell International 24 (2024): 61, 10.1186/s12935-024-03250-3. 38326804 PMC10851476

[17] L. Li , Q. Tang , J. Ge , et al., “METTL14 Promotes Lipid Metabolism Reprogramming and Sustains Nasopharyngeal Carcinoma Progression via Enhancing m(6)A Modification of ANKRD22 mRNA,” Clinical and Translational Medicine 14 (2024): e1766, 10.1002/ctm2.1766. 39021049 PMC11255023

[18] H. Liu , Y. Li , L. Tang , et al., “UBR5 Metabolically Reprograms Nasopharyngeal Carcinoma Cells to Promote Glycolysis and M2 Polarization via SPLUNC1 Signaling,” NPJ Precision Oncology 8 (2024): 252, 10.1038/s41698-024-00747-y. 39501021 PMC11538528

[19] C. Hu , S. Song , S. Zhao , Z. Xue , and X. Zhu , “METTL14 Contributes to the Progression of Nasopharyngeal Carcinoma Through Regulating the Stability of AOC1 mRNA,” Hereditas 161 (2024): 20, 10.1186/s41065-024-00317-z. 38956710 PMC11221105

[20] Y. M. Wang , Z. Y. Peng , L. Y. Zhang , et al., “N6‐Methyladenosine RNA Modification Landscape in the Occurrence and Recurrence of Nasopharyngeal Carcinoma,” World Journal of Oncology 13 (2022): 205–215, 10.14740/wjon1491. 36128587 PMC9451570

[21] D. Jian , Y. Wang , L. Jian , et al., “METTL14 Aggravates Endothelial Inflammation and Atherosclerosis by Increasing FOXO1 N6‐Methyladeosine Modifications,” Theranostics 10 (2020): 8939–8956, 10.7150/thno.45178. 32802173 PMC7415798

[22] Y. Zheng , Y. Li , X. Ran , et al., “Mettl14 Mediates the Inflammatory Response of Macrophages in Atherosclerosis Through the NF‐κB/IL‐6 Signaling Pathway,” Cellular and Molecular Life Sciences 79, no. 6 (2022): 311, 10.1007/s00018-022-04331-0. 35598196 PMC9124663

[23] Y. Hu , L. Lei , L. Jiang , et al., “LncRNA UCA1 Promotes Keratinocyte‐Driven Inflammation via Suppressing METTL14 and Activating the HIF‐1alpha/NF‐kappaB Axis in Psoriasis,” Cell Death and Disease 14 (2023): 279, 10.1038/s41419-023-05790-4. 37076497 PMC10115875

[24] Y. Chen , X. Miao , Y. Xiang , et al., “Qinzhu Liangxue Inhibits IL‐6‐Induced Hyperproliferation and Inflammation in HaCaT Cells by Regulating METTL14/SOCS3/STAT3 Axis,” Journal of Ethnopharmacology 317 (2023): 116809, 10.1016/j.jep.2023.116809. 37336334

[25] J. Tang , Z. Yu , J. Xia , et al., “METTL14‐Mediated m6A Modification of TNFAIP3 Involved in Inflammation in Patients With Active Rheumatoid Arthritis,” Arthritis & Rhematology 75 (2023): 2116–2129, 10.1002/art.42629. 37327357

[26] Z. Lu , H. Liu , N. Song , et al., “METTL14 Aggravates Podocyte Injury and Glomerulopathy Progression Through N(6)‐Methyladenosine‐Dependent Downregulating of Sirt1,” Cell Death & Disease 12 (2021): 881, 10.1038/s41419-021-04156-y. 34580283 PMC8476597

[27] T. Zhang , C. Ding , H. Chen , et al., “M(6)A mRNA Modification Maintains Colonic Epithelial Cell Homeostasis via NF‐kappaB‐Mediated Antiapoptotic Pathway,” Science Advances 8 (2022): eabl5723, 10.1126/sciadv.abl5723. 35333576 PMC8956260

[28] Y. Chen , Y. Wu , L. Fang , et al., “METTL14‐m6A‐FOXO3a Axis Regulates Autophagy and Inflammation in Ankylosing Spondylitis,” Clinical Immunology 257 (2023): 109838, 10.1016/j.clim.2023.109838. 37935312

[29] Z. Xie , W. Yu , G. Zheng , et al., “TNF‐Alpha‐Mediated m(6)A Modification of ELMO1 Triggers Directional Migration of Mesenchymal Stem Cell in Ankylosing Spondylitis,” Nature Communications 12 (2021): 5373, 10.1038/s41467-021-25710-4. PMC8433149 34508078

[30] L. Wang , H. Hui , K. Agrawal , et al., “M(6) A RNA Methyltransferases METTL3/14 Regulate Immune Responses to Anti‐PD‐1 Therapy,” EMBO Journal 39 (2020): e104514, 10.15252/embj.2020104514. 32964498 PMC7560214

[31] L. Dong , C. Chen , Y. Zhang , et al., “The Loss of RNA N(6)‐Adenosine Methyltransferase Mettl14 in Tumor‐Associated Macrophages Promotes CD8(+) T Cell Dysfunction and Tumor Growth,” Cancer Cell 39 (2021): 945, e910–957, 10.1016/j.ccell.2021.04.016. 34019807

[32] S. Kong , J. Zhang , L. Wang , et al., “Mechanisms of Low MHC I Expression and Strategies for Targeting MHC I With Small Molecules in Cancer Immunotherapy,” Cancer Letters 611 (2024): 217432, 10.1016/j.canlet.2024.217432. 39730087

[33] T. Y. Lin , J. S. Jia , W. R. Luo , et al., “ThermomiR‐377‐3p‐Induced Suppression of Cirbp Expression Is Required for Effective Elimination of Cancer Cells and Cancer Stem‐Like Cells by Hyperthermia,” Journal of Experimental & Clinical Cancer Research 43 (2024): 62, 10.1186/s13046-024-02983-3. 38419081 PMC10903011

[34] Y. J. Qin , T. Y. Lin , X. L. Lin , et al., “Loss of PDK4 Expression Promotes Proliferation, Tumorigenicity, Motility and Invasion of Hepatocellular Carcinoma Cells,” Journal of Cancer 11 (2020): 4397–4405, 10.7150/jca.43459. 32489458 PMC7255379

[35] J. Li , T. Y. Lin , L. Chen , et al., “miR‐19 Regulates the Expression of Interferon‐Induced Genes and MHC Class I Genes in Human Cancer Cells,” International Journal of Medical Sciences 17 (2020): 953–964, 10.7150/ijms.44377. 32308549 PMC7163354

[36] X. Li , X. Xu , Q. Zhang , M. Ling , X. Li , and X. Tan , “METTL14 Promotes Fibroblast‐Like Synoviocytes Activation via the LASP1/SRC/AKT Axis in Rheumatoid Arthritis,” American Journal of Physiology. Cell Physiology 324 (2023): C1089–C1100, 10.1152/ajpcell.00575.2022. 36878846

[37] J. Chen , X. Lin , J. He , et al., “Artemisitene Suppresses Rheumatoid Arthritis Progression via Modulating METTL3‐Mediated N6‐Methyladenosine Modification of ICAM2 mRNA in Fibroblast‐Like Synoviocytes,” Clinical and Translational Medicine 12 (2022): e1148, 10.1002/ctm2.1148. 36536495 PMC9763537

[38] Y. Liu , X. Wang , M. Huang , et al., “METTL3 Facilitates Kidney Injury Through Promoting IRF4‐Mediated Plasma Cell Infiltration via an m6A‐Dependent Manner in Systemic Lupus Erythematosus,” BMC Medicine 22 (2024): 511, 10.1186/s12916-024-03735-y. 39501302 PMC11539763

[39] W. J. Ni , H. Zhou , H. Lu , et al., “Genetic and Pharmacological Inhibition of METTL3 Alleviates Renal Fibrosis by Reducing EVL m6A Modification Through an IGF2BP2‐Dependent Mechanism,” Clinical and Translational Medicine 13 (2023): e1359, 10.1002/ctm2.1359. 37537731 PMC10400756

[40] X. Han , L. Liu , S. Huang , et al., “RNA m(6)A Methylation Modulates Airway Inflammation in Allergic Asthma via PTX3‐Dependent Macrophage Homeostasis,” Nature Communications 14 (2023): 7328, 10.1038/s41467-023-43219-w. PMC10643624 37957139

[41] F. Cao , G. Chen , Y. Xu , et al., “METTL14 Contributes to Acute Lung Injury by Stabilizing NLRP3 Expression in an IGF2BP2‐Dependent Manner,” Cell Death & Disease 15 (2024): 43, 10.1038/s41419-023-06407-6. 38218935 PMC10787837

[42] G. Dong , J. Yu , G. Shan , L. Su , N. Yu , and S. Yang , “N6‐Methyladenosine Methyltransferase METTL3 Promotes Angiogenesis and Atherosclerosis by Upregulating the JAK2/STAT3 Pathway via m6A Reader IGF2BP1,” Frontiers in Cell and Development Biology 9 (2021): 731810, 10.3389/fcell.2021.731810. PMC8689138 34950654

[43] W. Wu , X. Li , Z. Zhou , et al., “METTL14 Regulates Inflammation in Ulcerative Colitis via the lncRNA DHRS4‐AS1/miR‐206/A3AR Axis,” Cell Biology and Toxicology 40 (2024): 95, 10.1007/s10565-024-09944-8. 39528760 PMC11554827

[44] E. Shacter and S. A. Weitzman , “Chronic Inflammation and Cancer,” Oncology (Williston Park) 16 (2002): 217–229. 11866137

[45] L. M. Coussens and Z. Werb , “Inflammation and Cancer,” Nature 420 (2002): 860–867, 10.1038/nature01322. 12490959 PMC2803035

[46] S. I. Grivennikov , F. R. Greten , and M. Karin , “Immunity, Inflammation, and Cancer,” Cell 140 (2010): 883–899, 10.1016/j.cell.2010.01.025. 20303878 PMC2866629

[47] N. Singh , D. Baby , J. P. Rajguru , P. B. Patil , S. S. Thakkannavar , and V. B. Pujari , “Inflammation and Cancer,” Annals of African Medicine 18 (2019): 121–126, 10.4103/aam.aam_56_18. 31417011 PMC6704802

[48] H. Yin , X. Zhang , P. Yang , et al., “RNA m6A Methylation Orchestrates Cancer Growth and Metastasis via Macrophage Reprogramming,” Nature Communications 12 (2021): 1394, 10.1038/s41467-021-21514-8. PMC7925544 33654093

[49] C. Zhong , B. Tao , F. Yang , et al., “Histone Demethylase JMJD1C Promotes the Polarization of M1 Macrophages to Prevent Glioma by Upregulating miR‐302a,” Clinical and Translational Medicine 11 (2021): e424, 10.1002/ctm2.424. 34586733 PMC8473479

[50] J. He , M. Zhou , J. Yin , et al., “METTL3 Restrains Papillary Thyroid Cancer Progression via m(6)A/c‐Rel/IL‐8‐Mediated Neutrophil Infiltration,” Molecular Therapy 29 (2021): 1821–1837, 10.1016/j.ymthe.2021.01.019. 33484966 PMC8116572

[51] Z. Wang , J. Shang , Y. Qiu , et al., “Suppression of the METTL3‐m(6)A‐Integrin beta1 Axis by Extracellular Acidification Impairs T Cell Infiltration and Antitumor Activity,” Cell Reports 43 (2024): 113796, 10.1016/j.celrep.2024.113796. 38367240

[52] Q. Xi , G. Yang , X. He , et al., “M(6)A‐Mediated Upregulation of lncRNA TUG1 in Liver Cancer Cells Regulates the Antitumor Response of CD8(+) T Cells and Phagocytosis of Macrophages,” Adv Sci (Weinh) 11 (2024): e2400695, 10.1002/advs.202400695. 38981064 PMC11425850

[53] X. Mu , Q. Zhao , W. Chen , et al., “IL‐37 Confers Anti‐Tumor Activity by Regulation of m6A Methylation,” Frontiers in Oncology 10 (2020): 526866, 10.3389/fonc.2020.526866. 33489865 PMC7821743

[54] D. E. Johnson , R. A. O'Keefe , and J. R. Grandis , “Targeting the IL‐6/JAK/STAT3 Signalling Axis in Cancer,” Nature Reviews. Clinical Oncology 15 (2018): 234–248, 10.1038/nrclinonc.2018.8. PMC5858971 29405201

[55] Q. Chang , E. Bournazou , P. Sansone , et al., “The IL‐6/JAK/Stat3 Feed‐Forward Loop Drives Tumorigenesis and Metastasis,” Neoplasia 15 (2013): 848–862, 10.1593/neo.13706. 23814496 PMC3689247

[56] C. H. Hung , S. Y. Wu , C. D. Yao , et al., “Defective N‐Glycosylation of IL6 Induces Metastasis and Tyrosine Kinase Inhibitor Resistance in Lung Cancer,” Nature Communications 15 (2024): 7885, 10.1038/s41467-024-51831-7. PMC11385228 39251588

[57] S. T. Orange , J. Leslie , M. Ross , D. A. Mann , and H. Wackerhage , “The Exercise IL‐6 Enigma in Cancer,” Trends in Endocrinology and Metabolism 34 (2023): 749–763, 10.1016/j.tem.2023.08.001. 37633799

[58] L. C. Chan , C. W. Li , W. Xia , et al., “IL‐6/JAK1 Pathway Drives PD‐L1 Y112 Phosphorylation to Promote Cancer Immune Evasion,” Journal of Clinical Investigation 129 (2019): 3324–3338, 10.1172/JCI126022. 31305264 PMC6668668

[59] Q. Liu , S. Yu , A. Li , H. Xu , X. Han , and K. Wu , “Targeting Interlukin‐6 to Relieve Immunosuppression in Tumor Microenvironment,” Tumor Biology 39 (2017): 1010428317712445, 10.1177/1010428317712445. 28639898

[60] P. Sadhukhan , M. Feng , E. Illingworth , et al., “YAP1 Induces Bladder Cancer Progression and Promotes Immune Evasion Through IL‐6/STAT3 Pathway and CXCL Deregulation,” Journal of Clinical Investigation 135 (2024): e171164, 10.1172/JCI171164. 39630608 PMC11735109

[61] B. R. Choi , K. R. Johnson , D. Maric , and D. B. McGavern , “Monocyte‐Derived IL‐6 Programs Microglia to Rebuild Damaged Brain Vasculature,” Nature Immunology 24 (2023): 1110–1123, 10.1038/s41590-023-01521-1. 37248420 PMC11531796

[62] R. Catar , J. Witowski , N. Zhu , et al., “IL‐6 Trans‐Signaling Links Inflammation With Angiogenesis in the Peritoneal Membrane,” J Am Soc Nephrol 28 (2017): 1188–1199, 10.1681/ASN.2015101169. 27837150 PMC5373439

[63] M. Zhang , H. Yang , L. Wan , et al., “Single‐Cell Transcriptomic Architecture and Intercellular Crosstalk of Human Intrahepatic Cholangiocarcinoma,” Journal of Hepatology 73 (2020): 1118–1130, 10.1016/j.jhep.2020.05.039. 32505533

[64] F. Yang , Z. He , H. Duan , et al., “Synergistic Immunotherapy of Glioblastoma by Dual Targeting of IL‐6 and CD40,” Nature Communications 12 (2021): 3424, 10.1038/s41467-021-23832-3. PMC8187342 34103524

[65] M. Muckenhuber , K. Mengrelis , A. M. Weijler , et al., “IL‐6 Inhibition Prevents Costimulation Blockade‐Resistant Allograft Rejection in T Cell‐Depleted Recipients by Promoting Intragraft Immune Regulation in Mice,” Nature Communications 15 (2024): 4309, 10.1038/s41467-024-48574-w. PMC11148062 38830846

[66] F. Hu , D. Song , Y. Yan , et al., “IL‐6 Regulates Autophagy and Chemotherapy Resistance by Promoting BECN1 Phosphorylation,” Nature Communications 12 (2021): 3651, 10.1038/s41467-021-23923-1. PMC8206314 34131122

[67] Y. S. Weng , H. Y. Tseng , Y. A. Chen , et al., “MCT‐1/miR‐34a/IL‐6/IL‐6R Signaling Axis Promotes EMT Progression, Cancer Stemness and M2 Macrophage Polarization in Triple‐Negative Breast Cancer,” Molecular Cancer 18 (2019): 42, 10.1186/s12943-019-0988-0. 30885232 PMC6421700

[68] M. Zhuang , X. Ding , W. Song , et al., “Correlation of IL‐6 and JAK2/STAT3 Signaling Pathway With Prognosis of Nasopharyngeal Carcinoma Patients,” Aging (Albany NY) 13 (2021): 16667–16683, 10.18632/aging.203186. 34165442 PMC8266356

[69] J. Dong , J. Chen , Q. Li , and S. Qiu , “Knockdown of FKBP3 Suppresses Nasopharyngeal Carcinoma Cell Growth, Invasion and Migration, Deactivated NF‐kappaB/IL‐6 Signaling Pathway Through Inhibiting Histone Deacetylase 2 Expression,” Chinese Journal of Physiology 66 (2023): 85–92, 10.4103/cjop.CJOP-D-22-00075. 37082996

[70] X. Zhang , J. Yang , Z. Bian , D. Shi , and Z. Cao , “Long Noncoding RNA DANCR Promotes Nasopharyngeal Carcinoma Progression by Interacting With STAT3, Enhancing IL‐6/JAK1/STAT3 Signaling,” Biomedicine & Pharmacotherapy 113 (2019): 108713, 10.1016/j.biopha.2019.108713. 30849642

[71] S. Ghose , S. Roy , V. Ghosh , et al., “The Plasma EBV DNA Load With IL‐6 and VEGF Levels as Predictive and Prognostic Biomarker in Nasopharyngeal Carcinoma,” Virology Journal 21 (2024): 224, 10.1186/s12985-024-02473-0. 39304953 PMC11414088

[72] N. Kerur , M. V. Veettil , N. Sharma‐Walia , et al., “IFI16 Acts as a Nuclear Pathogen Sensor to Induce the Inflammasome in Response to Kaposi Sarcoma‐Associated Herpesvirus Infection,” Cell Host & Microbe 9 (2011): 363–375, 10.1016/j.chom.2011.04.008. 21575908 PMC3113467

[73] H. Cai , L. Yan , N. Liu , M. Xu , and H. Cai , “IFI16 Promotes Cervical Cancer Progression by Upregulating PD‐L1 in Immunomicroenvironment Through STING‐TBK1‐NF‐kB Pathway,” Biomedicine & Pharmacotherapy 123 (2020): 109790, 10.1016/j.biopha.2019.109790. 31896065

[74] L. T. Ong , W. C. Lee , S. Ma , et al., “IFI16‐Dependent STING Signaling Is a Crucial Regulator of Anti‐HER2 Immune Response in HER2+ Breast Cancer,” Proceedings of the National Academy of Sciences of The United States of America 119 (2022): e2201376119, 10.1073/pnas.2201376119. 35878022 PMC9351446

[75] Y. Azumi , Y. I. Koma , S. Tsukamoto , et al., “IFI16 Induced by Direct Interaction Between Esophageal Squamous Cell Carcinomas and Macrophages Promotes Tumor Progression via Secretion of IL‐1alpha,” Cells 12 (2023): 2603, 10.3390/cells12222603. 37998338 PMC10670642

[76] Q. Yan , J. Zhou , Z. Wang , et al., “NAT10‐Dependent N(4)‐Acetylcytidine Modification Mediates PAN RNA Stability, KSHV Reactivation, and IFI16‐Related Inflammasome Activation,” Nature Communications 14 (2023): 6327, 10.1038/s41467-023-42135-3. PMC10564894 37816771

[77] N. L. Ka , G. Y. Lim , S. Hwang , S. S. Kim , and M. O. Lee , “IFI16 Inhibits DNA Repair That Potentiates Type‐I Interferon‐Induced Antitumor Effects in Triple Negative Breast Cancer,” Cell Reports 37 (2021): 110138, 10.1016/j.celrep.2021.110138. 34936865

[78] A. Iannucci , V. Caneparo , S. Raviola , et al., “Toll‐Like Receptor 4‐Mediated Inflammation Triggered by Extracellular IFI16 Is Enhanced by Lipopolysaccharide Binding,” PLoS Pathogens 16 (2020): e1008811, 10.1371/journal.ppat.1008811. 32903274 PMC7505474

[79] Y. Kobayashi , M. A. Bustos , Y. Hayashi , Q. Yu , and D. Hoon , “Interferon‐Induced Factor 16 Is Essential in Metastatic Melanoma to Maintain STING Levels and the Immune Responses Upon IFN‐Gamma Response Pathway Activation,” Journal for Immunotherapy of Cancer 12 (2024): e009590, 10.1136/jitc-2024-009590. 39424359 PMC11492949

[80] J. Mazibrada , M. De Andrea , M. Ritta , et al., “In Vivo Growth Inhibition of Head and Neck Squamous Cell Carcinoma by the Interferon‐Inducible Gene IFI16,” Cancer Letters 287 (2010): 33–43, 10.1016/j.canlet.2009.05.035. 19553003

[81] X. L. Shi , J. Yang , N. Mao , et al., “Nutlin‐3‐Induced Redistribution of Chromatin‐Bound IFI16 in Human Hepatocellular Carcinoma Cells In Vitro Is Associated With p53 Activation,” Acta Pharmacologica Sinica 36 (2015): 252–258, 10.1038/aps.2014.106. 25544361 PMC4326783

[82] B. H. Sliker , B. T. Goetz , R. Barnes , et al., “HLA‐B Influences Integrin Beta‐1 Expression and Pancreatic Cancer Cell Migration,” Experimental Cell Research 390 (2020): 111960, 10.1016/j.yexcr.2020.111960. 32194036 PMC7182497

[83] M. Gomez‐Herranz , J. Faktor , M. Yebenes Mayordomo , et al., “Emergent Role of IFITM1/3 Towards Splicing Factor (SRSF1) and Antigen‐Presenting Molecule (HLA‐B) in Cervical Cancer,” Biomolecules 12 (2022): 12081090, 10.3390/biom12081090. PMC9405601 36008984

[84] Z. Lu , H. Chen , X. Jiao , et al., “Germline HLA‐B Evolutionary Divergence Influences the Efficacy of Immune Checkpoint Blockade Therapy in Gastrointestinal Cancer,” Genome Medicine 13 (2021): 175, 10.1186/s13073-021-00997-6. 34732240 PMC8567649

[85] T. Michelakos , F. Kontos , T. Kurokawa , et al., “Differential Role of HLA‐A and HLA‐B, C Expression Levels as Prognostic Markers in Colon and Rectal Cancer,” Journal for Immunotherapy of Cancer 10 (2022): 004115, 10.1136/jitc-2021-004115. PMC8919449 35277460

[86] W. J. Raab , A. Mazzocchi , P. Radice , et al., “A Microsatellite in the Coding Sequence of HLA‐A/B Is a Mutation Hotspot in Colon Cancer With Microsatellite Instability,” Gastroenterology 162 (2022): 960–e963, 10.1053/j.gastro.2021.10.006. 34653421 PMC8881331

[87] J. Prakash and Y. Shaked , “The Interplay Between Extracellular Matrix Remodeling and Cancer Therapeutics,” Cancer Discovery 14 (2024): 1375–1388, 10.1158/2159-8290.cd-24-0002. 39091205 PMC11294818

[88] Y. Chen , Z. Lu , C. Qi , et al., “N(6)‐Methyladenosine‐Modified TRAF1 Promotes Sunitinib Resistance by Regulating Apoptosis and Angiogenesis in a METTL14‐Dependent Manner in Renal Cell Carcinoma,” Molecular Cancer 21 (2022): 111, 10.1186/s12943-022-01549-1. 35538475 PMC9087993

[89] Q. Kang , X. Zhu , D. Ren , et al., “Adipose METTL14‐Elicited N(6) ‐Methyladenosine Promotes Obesity, Insulin Resistance, and NAFLD Through Suppressing β Adrenergic Signaling and Lipolysis,” Adv Sci (Weinh) 10 (2023): e2301645, 10.1002/advs.202301645. 37526326 PMC10558699

[90] S. W. Tsao , C. M. Tsang , and K. W. Lo , “Epstein‐Barr Virus Infection and Nasopharyngeal Carcinoma,” Philosophical Transactions of the Royal Society of London. Series B, Biological Sciences 372 (2017): 0270, 10.1098/rstb.2016.0270. PMC5597737 28893937

[91] C. Y. Lim , G. W. Y. Ng , C. K. Goh , et al., “Impact of High‐Risk EBV Strains on Nasopharyngeal Carcinoma Gene Expression,” Oral Oncology 157 (2024): 106941, 10.1016/j.oraloncology.2024.106941. 39024697

[92] A. R. Marquitz and N. Raab‐Traub , “The Role of miRNAs and EBV BARTs in NPC,” Seminars in Cancer Biology 22 (2012): 166–172, 10.1016/j.semcancer.2011.12.001. 22178394 PMC3340885

[93] S. Barth , G. Meister , and F. A. Grässer , “EBV‐Encoded miRNAs,” Biochimica et Biophysica Acta 1809 (2011): 631–640, 10.1016/j.bbagrm.2011.05.010. 21640213

