# Supplementary material for: METTL14 Regulates the Expression of Genes Related to Interferon, Interleukin and MHC Class I in Nasopharyngeal Carcinoma Cells
Source: Cancer Med. 2025 Nov 28;14(23):e71371. doi: 10.1002/cam4.71371 (PMC12662788; doi:10.1002/cam4.71371)
Supplement: Supplementary file 1 — Data S1: cam471371‐sup‐0001‐TableS1‐S6‐FigureS1‐S7.docx. [file CAM4-14-e71371-s001.docx]

**Supplementary table 1 Oligonucleotide sequence**

| shRNA-NC | CGTGATCTTCACCGACAAGAT |
| --- | --- |
| shMETTL14-A | GCTGACAGATTTGAAGAATAT |
| shMETTL14-B | TGGACTTGGGATGATATTAT |

| **Supplementary table 2 Primers for qRT-PCR analysis of METTL14** | | |
| --- | --- | --- |
| **Gene** | **Forward Primer (5'-3')** | **Reverse Primer (5'-3')** |
| GAPDH | ACCCAGAAGACTGTGGATGG | TCTAGACGGCAGGTCAGGTC |
| METTL14 | GAGTGTGTTTACGAAAATGGGGT | CCGTCTGTGCTACGCTTCA |

| **Supplementary table 3 Primers for qRT-PCR analysis of TNF-related genes** | | |
| --- | --- | --- |
| **Gene** | **Forward Primer (5'-3')** | **Reverse Primer (5'-3')** |
| TRAIL | CCCAATGACGAAGAGAGTATGA | GGAATAGATGTAGTAAAACCCT |
| TNFAIP3 | TCCTCAGGCTTTGTATTTGAGC | TGTGTATCGGTGCATGGTTTTA |
| TNFRSF12A | TCTGAGCCTGACCTTCGTGCTG | GGCACATTGTCACTGGATCAGC |
| TNFRSF25 | CCGTCCAGTTGGTGGGTAAC | CCATCACGTCGTAGAGCTGC |
| TNFRSF9 | AGCTGTTACAACATAGTAGCCAC | GGACAGGGACTGCAAATCTGAT |
| TNFSF10 | TGCGTGCTGATCGTGATCTTC | GCTCGTTGGTAAAGTACACGTA |
| TNFSF14 | AGAAATCGTGTGTTTGCCTCA | TCCAGTCCTATTGAATGTGGGA |
| TNFSF15 | GGTCTCTTGCTGTTGCTGATGG | TTGACCTCGTGAGACCTTCGCT |
| TNFSF9 | GGCTGGAGTCTACTATGTCTTCT | ACCTCGGTGAAGGGAGTCC |
| TRAF1 | TCCTGTGGAAGATCACCAATGT | GCAGGCACAACTTGTAGCC |
| TRAF2 | TCCCTGGAGTTGCTACAGC | AGGCGGAGCACAGGTACTT |
| TRAF3 | CAGACTAACCCGCCGCTAAAG | GATGCTCTCTTGACACGCTGT |

| **Supplementary table 4 Primers for qRT-PCR analysis of interferon-regulated genes** | | |
| --- | --- | --- |
| **Gene** | **Forward Primer (5'-3')** | **Reverse Primer (5'-3')** |
| AIM2 | TCAAGCTGAAATGAGTCCTGC | CTTGGGTCTCAAACGTGAAGG |
| B2M | GAGGCTATCCAGCGTACTCCA | CGGCAGGCATACTCATCTTTT |
| GBP1 | GCCTTCGGTGTATTTCCCTG | GCAACTGGACCCTGTCGTT |
| IFI16 | AGACTGAAGACTGAACCTGAAGA | GAACCCATTGCGGCAAACATA |
| IFI27 | TGCTCTCACCTCATCAGCAGT | CACAACTCCTCCAATCACAACT |
| IFI35 | GTGGACGTTCGGGAGCTAC | ACTGGCCGATTTGGCACAG |
| IFI44L | AGCCGTCAGGGATGTACTATAAC | AGGGAATCATTTGGCTCTGTAGA |
| IFI6 | GGTCTGCGATCCTGAATGGG | TCACTATCGAGATACTTGTGGGT |
| IFIT1 | TTGATGACGATGAAATGCCTGA | CAGTCACCAGACTCCTCAC |
| IFIT2 | AAGCACCTCAAAGGGCAAAAC | TCGGCCCATGTGATAGTAGAC |
| IFIT3 | ACTCTTTTCCTCCCAGAGGG | GGGCTGAGCAGTTCAGAAA |
| IFITM1 | CCAAGGTCCACCGTGATTAAC | ACCAGTTCAAGAAGAGGGTGTT |
| IRF1 | TCAAGTTCCGTTTGCAGTACC | GCACTGACTCGTACAGCATGG |
| IRF5 | GCTGTGCCCTTAACAAGAGC | TGCACCAAAAGAGTAATCCTCAG |
| IRF7 | CCCACGCTATACCATCTACCT | GATGTCGTCATAGAGGCTGTTG |
| ISG20 | CTCGTTGCAGCCTCGTGAA | CGGGTTCTGTAATCGGTGATCTC |
| PSMB10 | TCCTTCGAGAACTGCCAAAGA | ATCGTTAGTGGCTCGCGTATC |
| PSMB8 | CACGCTCGCCTTCAAGTTC | AGGCACTAATGTAGGACCCAG |
| IFIH1 | TCGAATGGGTATTCCACAGACG | GTGGCGACTGTCCTCTGAA |
| IFNL2 | TCGCTTCTGCTGAAGGACTGCA | CCTCCAGAACCTTCAGCGTCAG |
| IRF6 | CCCCAGGCACCTATACAGC | TCCTTCCCACGGTACTGAAAC |
| IRF9 | GCCCTACAAGGTGTATCAGTTG | TGCTGTCGCTTTGATGGTACT |
| IRS1 | ACAAACGCTTCTTCGTACTGC | AGTCAGCCCGCTTGTTGATG |
| PSMA2 | GAGCGCGGGTACAGCTTTT | ACCACACCATTTGCAGCTTTA |
| PSMA3 | GCTCAATCGGCACTGGGTAT | ACCTGCTACTGCCATTCCAAC |
| PSMB2 | ATCCTCGACCGATACTACACAC | GAACACTGAAGGTTGGCAGAT |
| PSMB5 | AGGAACGCATCTCTGTAGCAG | AGGGCCTCTCTTATCCCAGC |
| PSMB6 | GGCTACCTTACTAGCTGCTCG | GATTGGCGATGTAGGACCCAG |
| PSMC6 | ATATGTTGTGGGTTGTCGTCG | TCTGTTCTGATAGCCCTCCAAT |
| PSMD1 | TCCGAGTCCGTAGACAAAATAGA | CCACACATTGTTTGGTGTAGTGA |
| SOCS2 | TTAAAAGAGGCACCAGAAGGAAC | AGTCGATCAGATGAACCACACT |

| **Supplementary table 5 Primers for qRT-PCR analysis of interleukin-related genes** | | |
| --- | --- | --- |
| **Gene** | **Forward Primer (5'-3')** | **Reverse Primer (5'-3')** |
| GALT | CTGGGCTAGGGCATGAACTG | CTGGGACTCCAAGTGCAAGA |
| IL11 | TACTCCCAGGTCACATTCCGT | GGGTAGCTGTCTGTTACCTTGG |
| IL11RA | GGACCACAACCTGGATTCCCTG | AGTAGGTCCGCTCGCAGCCTT |
| IL1A | AGATGCCTGAGATACCCAAAACC | CCAAGCACACCCAGTAGTCT |
| IL1B | CAGCTACGAATCTCCGACCAC | GGCAGGGAACCAGCATCTTC |
| IL1F8 | CTGTGCGAGTGTACCGGATG | ATCCCCACATGACTTCCTCTT |
| IL20RB | GGCCACTGTGCCATACAAC | TCTTTGGTGATCTCCATCCCA |
| IL32 | AGCTGGAGGACGACTTCAAA | AGAGCAGCAGAAACTCTGGA |
| IL7 | TGAAGGTAAAGATGGCAAACAA | CAATTTCTTTCATGCTGTCCAA |
| IL7R | CCCTCGTGGAGGTAAAGTGC | CCTTCCCGATAGACGACACTC |
| CCL5 | CCAGCAGTCGTCTTTGTCAC | CTCTGGGTTGGCACACACTT |
| CXCL10 | GTGGCATTCAAGGAGTACCTC | TGATGGCCTTCGATTCTGGATT |
| CXCL2 | GGCAGAAAGCTTGTCTCAACCC | CTCCTTCAGGAACAGCCACCAA |
| IL10RB | TACCACCTCCCGAAAATGTCA | CCCAGTCTGAATGCTCATCTG |
| IL12A | ATGGCCCTGTGCCTTAGTAGT | AGCTTTGCATTCATGGTCTTGA |
| IL15 | TTGGGAACCATAGATTTGTGCAG | GGGTGAACATCACTTTCCGTAT |
| IL17D | TGAGCAGGCGCGCAACGCGA | GCAGTAGGCTTCAGGCAGGTAC |
| IL1RL1 | GCTCCTCTTAGCCGCAGCATTA | TCCCTTGATTCCCAGGTAAACCA |
| IL22RA1 | CACCCCAGACACGGTCTACA | GGCTTGAGGGTAGTGTGCTG |
| IL33 | GTGACGGTGTTGATGGTAAGAT | AGCTCCACAGAGTGTTCCTTG |
| IL6R | CCCCTCAGCAATGTTGTTTGT | CTCCGGGACTGCTAACTGG |
| IL8 | ACTGAGAGTGATTGAGAGTGGAC | AACCCTCTGCACCCAGTTTTC |
| NFKB2 | ATGGAGAGTTGCTACAACCCA | CTGTTCCACGATCACCAGGTA |
| IL6 | ACTCACCTCTTCAGAACGAATTG | CCATCTTTGGAAGGTTCAGGTTG |

| **Supplementary table 6 Primers for qRT-PCR analysis of MHC class I genes** | | |
| --- | --- | --- |
| **Gene** | **Forward Primer (5'-3')** | **Reverse Primer (5'-3')** |
| HLA-B | CAGTTCGTGAGGTTCGACAG | CAGCCGTACATGCTCTGGA |
| HLA-E | TTCCGAGTGAATCTGCGGAC | GTCGTAGGCGAACTGTTCATAC |
| HLA-F | TGGCCCTGACCGATACTTG | GCAGGAATTGCGTGTCGTC |
| HLA-G | GAAGAGGAGACACGGAACACCA | TCGCAGCCAATCATCCACTGGA |
| TAP1 | TGCCCCGCATATTCTCCCT | CACCTGCGTTTTCGCTCTTG |
| HLA-A | AAAAGGAGGGAGTTACACTCAGG | GCTGTGAGGGACACATCAGAG |
| HLA-C | GGAGACACAGAAGTACAAGCGC | ACATCCTCTGGAGGGTGTGAGA |


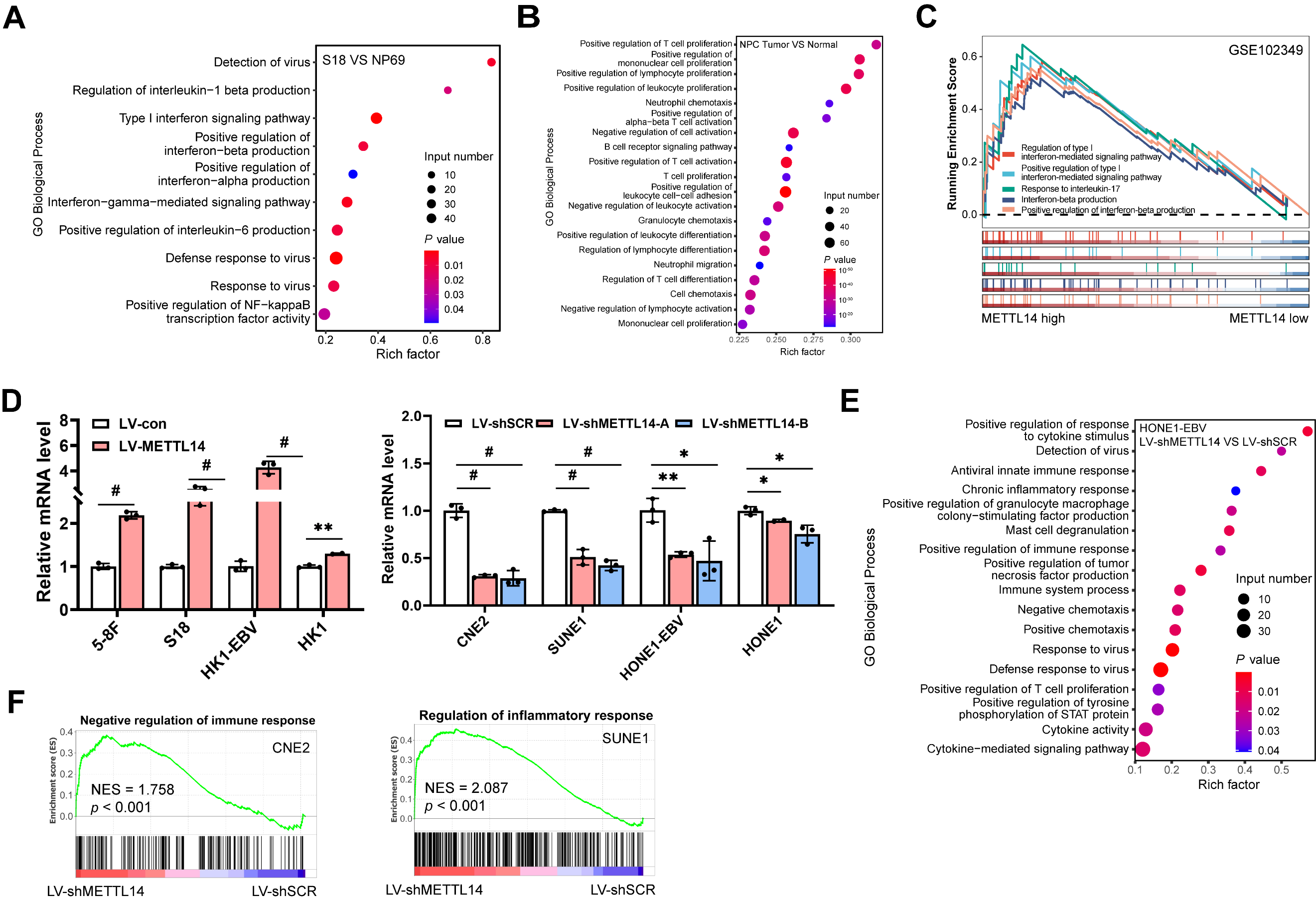


**Supplementary figure 1.** **A comprehensive analysis of immune- and inflammation-related gene expression differences in shMETTL14- or METTL14-expressing NPC cells.**

**(A)** GO analysis of up- and down-regulated related with immune- and inflammation-related genes in S18 and NP69 cells.

**(B)** GO analysis of up-and down-regulated immune- and inflammation-related genes in NPC tumors and normal tissues.

**(C)** GSEA result for functional enrichment analysis immune response-related genes set of some pathways in METTL14-high group and METTL14-low group using the GEO(GSE102349) database.

**(D)** METTL14 expression was analyzed by qRT-PCR in NPC cells which were transduced with lentivirus expressing METTL14 or shMETTL14.

**(E)** GO analysis of up- and down-regulated related with immune- and inflammation-related genes in shSCR-expressing and shMETTL14-expressing HONE1-EBV cells.

**(F)** GSEA plots indicate enriched gene sets of immune- and inflammation-associated genes in shSCR-expressing and shMETTL14-expressing CNE2 and SUNE1 cells. (NES: Normalized Enrichment Score)


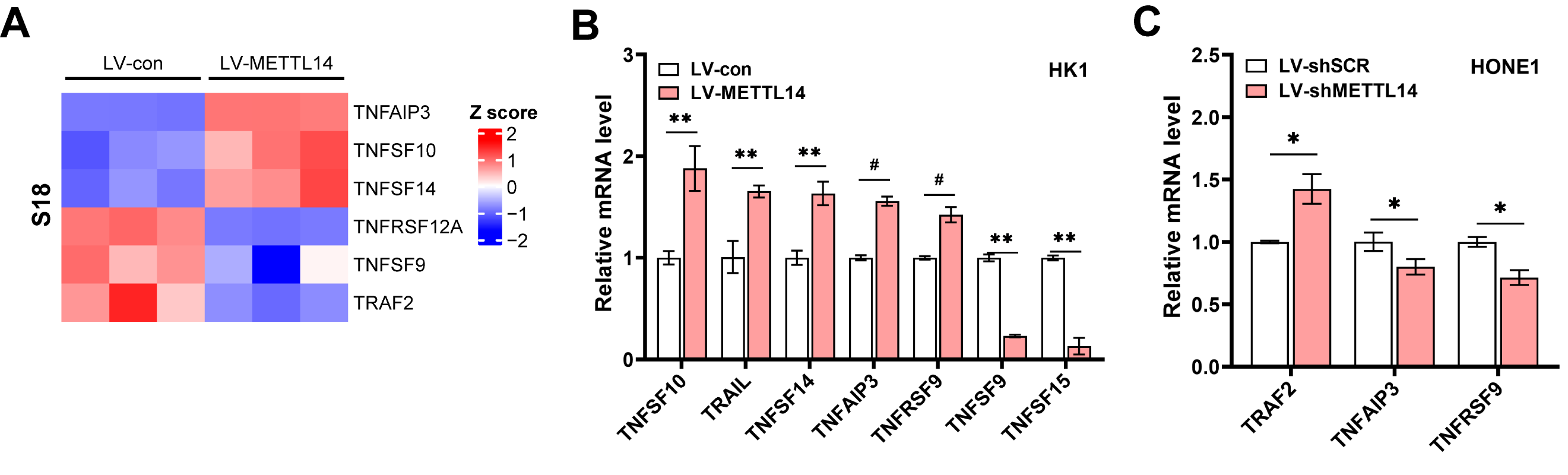
**Supplementary figure 2. Analysis of differentially expressed TNF-related genes in NPC cells.**

**(A)** Heatmap was used for visualization of the differentially expressed TNF-related genes between vector-expressing and METTL14-expressing S18 cells.

**(B-C)** qRT-PCR measurement of TNF-regulated gene expression in HK1 METTL14-expressing and HONE1 shMETTL14-expressing cells.


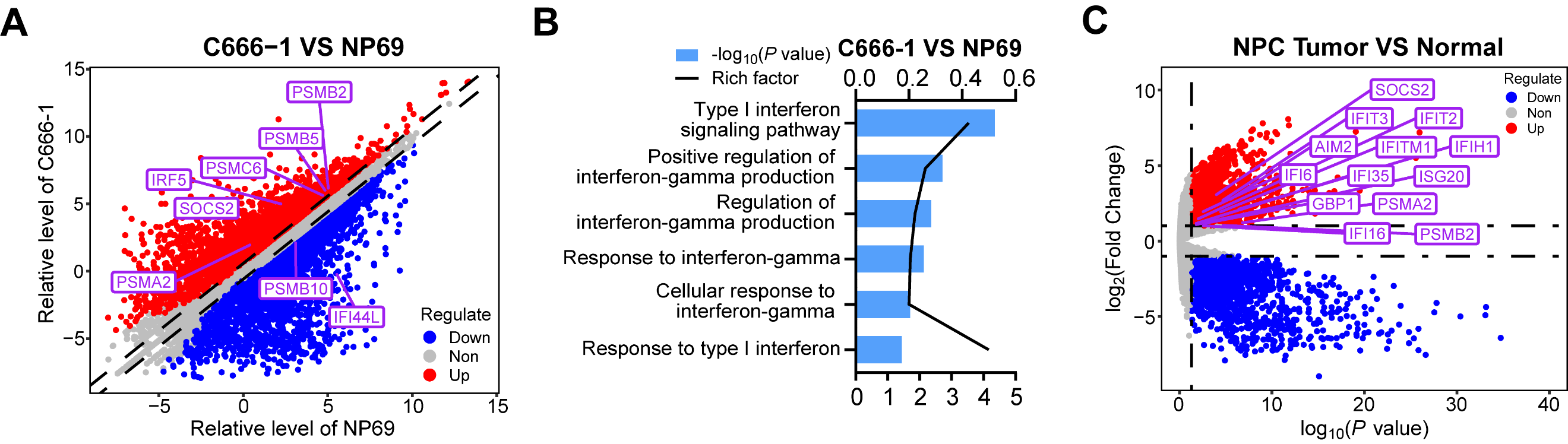


**Supplementary figure 3. Analysis of differentially expressed IFN-related genes in NPC cells and NPC tumors.**

**(A)** Scatter plots of the effect of expressed IFN-related genes in C666-1 cells and NP69 cells. The axes show gene expression in C666-1 cells and NP69 cells.

**(B)** The histogram of GO term enrichment analysis for IFN-related genes enriched in DEGs, with the significance of enrichment in C666-1 and NP69 cells

**(C)** Volcano plot of differentially expressed IFN-related genes in NPC tumors and normal tissues.


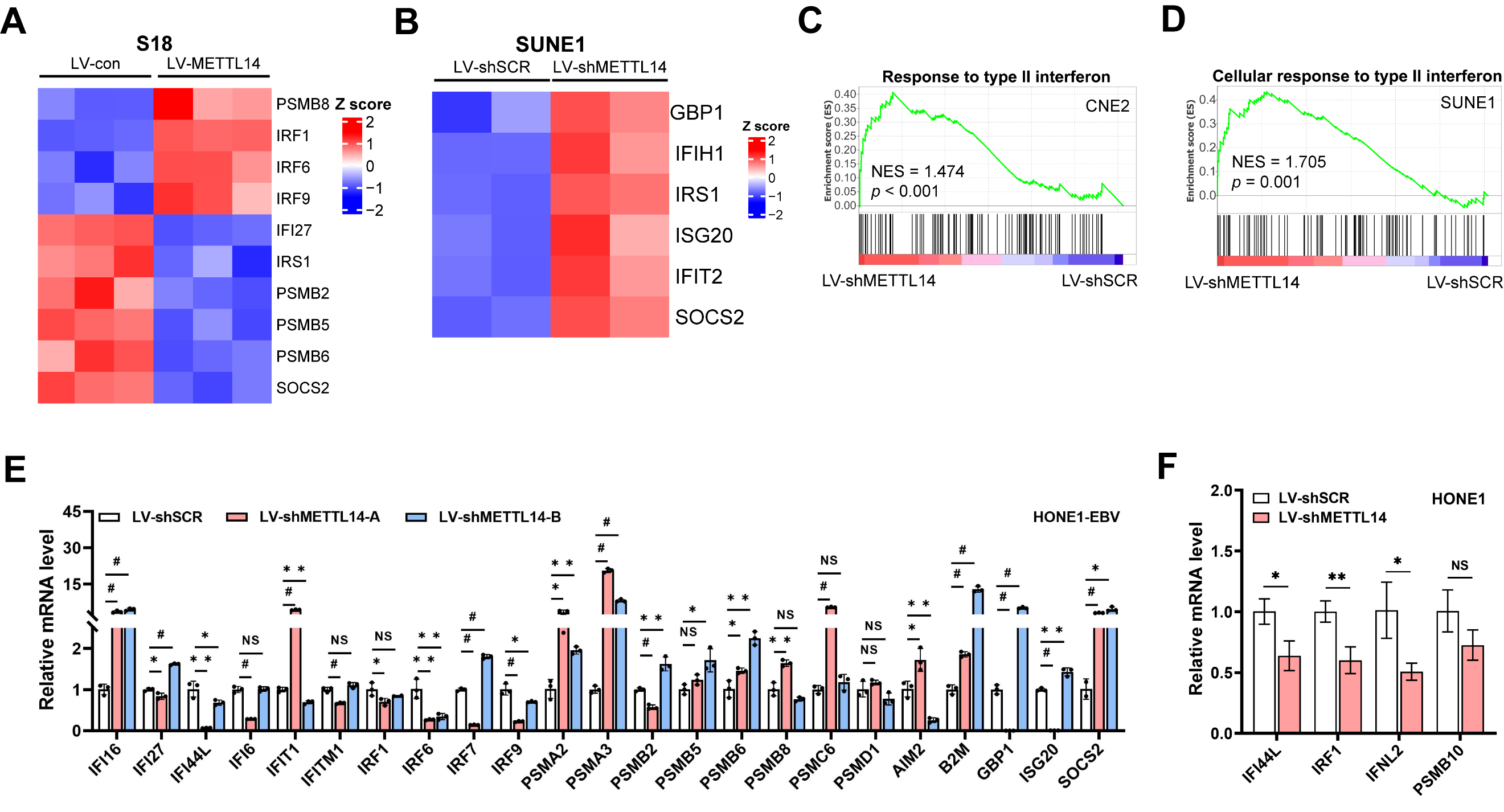


**Supplementary figure 4. Induction of IFN-regulated gene expression following METT14-** **or** **shMETTL14-expressing in NPC cells.**

**(A)** Heatmap representation of differentially expressed IFN-related genes between vector-expressing and METTL14-expressing S18 cells.

**(B)** Heatmap shows the differentially expressed IFN-related genes between shSCR- and shMETTL14-expressing SUNE1 cells.

**(C-D)**GSEA plot showing enrichment of IFN-like genes in shSCR- and shMETTL14-expressing CNE2(C) and SUNE1(D) cells.

**(E-F)** IFN-induced gene expression analyzed by qRT-PCR in HONE1-EBV and HONE1 cells expressing shMETTL14.


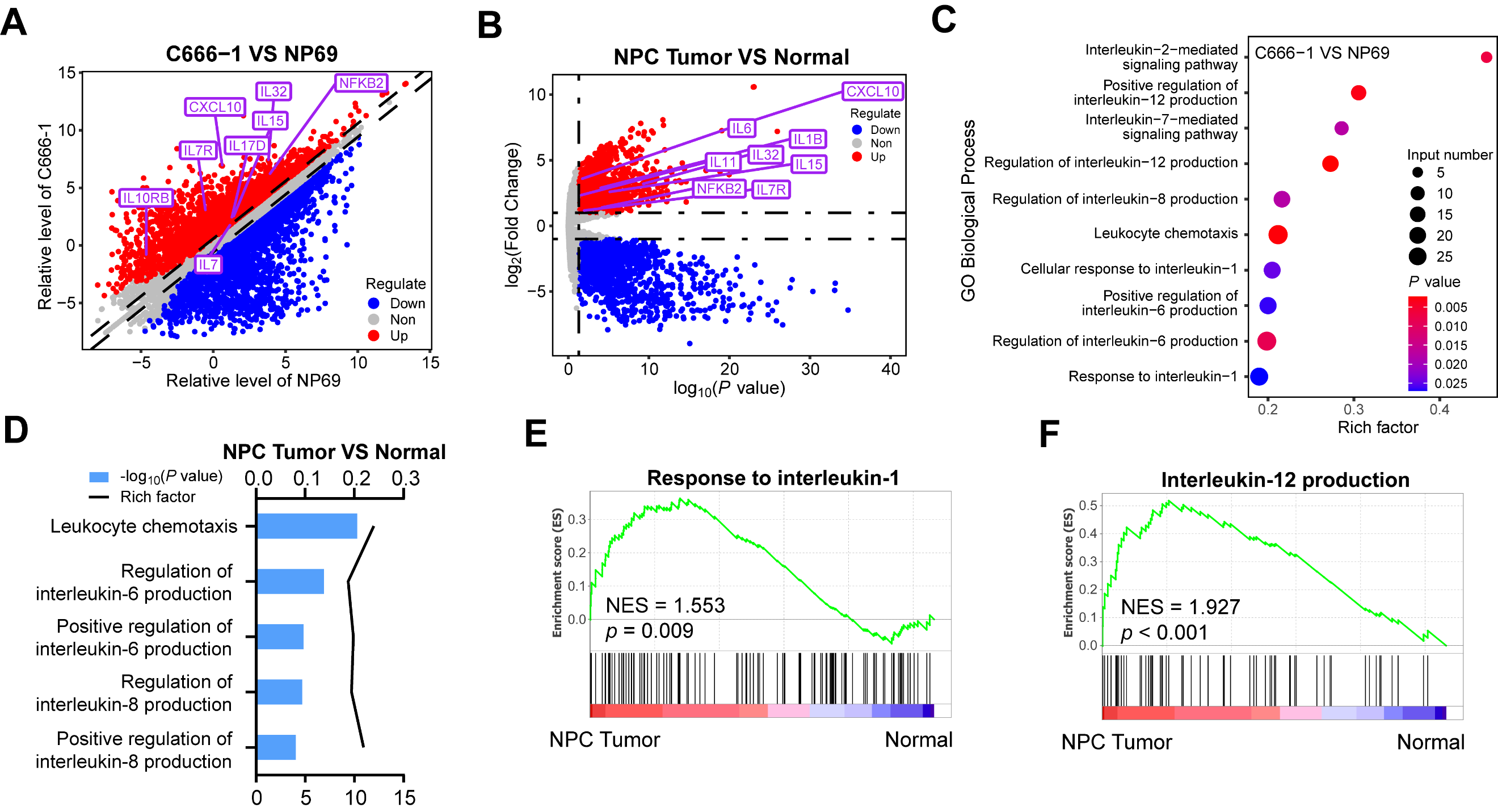


**Supplementary figure 5. Analysis of differentially expressed IL-related genes in NPC cells and NPC tumors.**

**(A)** Scatter plots analysis of the expression levels of IL-related genes in C666-1 cells and NP69 cells.

**(B)** Volcano plot illustrating the differential expression of IL-related genes in both tumors and normal tissues of NPC.

**(C)** GO analysis of up- and down-regulated IL-related genes in C666-1 cells and NP69 cells.

**(D)** Histogram of GO term enrichment analysis according to IL-related genes in NPC tumors and normal tissues.

**(E-F)** GSEA plot of enrichment of IL-related genes in NPC tumors and normal tissues.


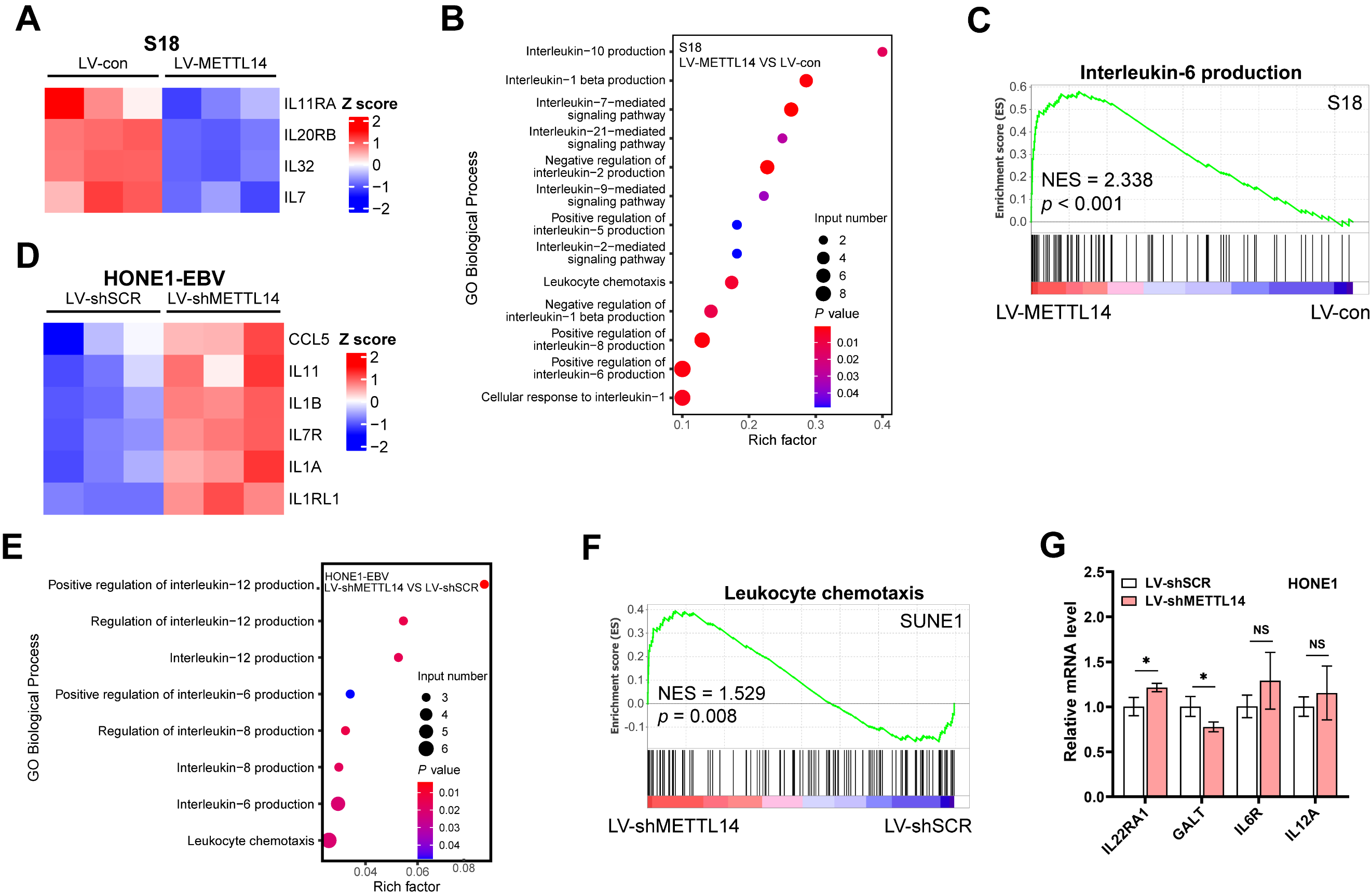


**Supplementary figure 6. The expression of IL-related genes following METT14- or shMETTL14-expressing in NPC cells.**

**(A)** Heatmap shows differentially expressed IL-related genes between vector-expressing and METTL14-expressing S18 cells.

**(B)** GO analysis of up-and down-regulated genes associated with IL in vector-expressing and METTL14-expressing S18 cells.

**(C)** GSEA plot for enrichment of IL-related genes in either vector- or METTL14-expressing S18 cells.

**(D)** Heatmap of the differential expression of IL-related genes in shSCR- and shMETTL14-expressing HONE1-EBV cells.

**(E)** GO analysis of up- and downregulated genes associated with IL in shSCR- and shMETTL14-expressing HONE1-EBV cells.

**(F)** GSEA plot demonstrating enrichment of IL-related genes in shSCR- and shMETTL14-expressing SUNE1 cells.

**(G)** IL-related genes expression analyzed by qRT-PCR in HONE1 cells expressing shMETTL14.


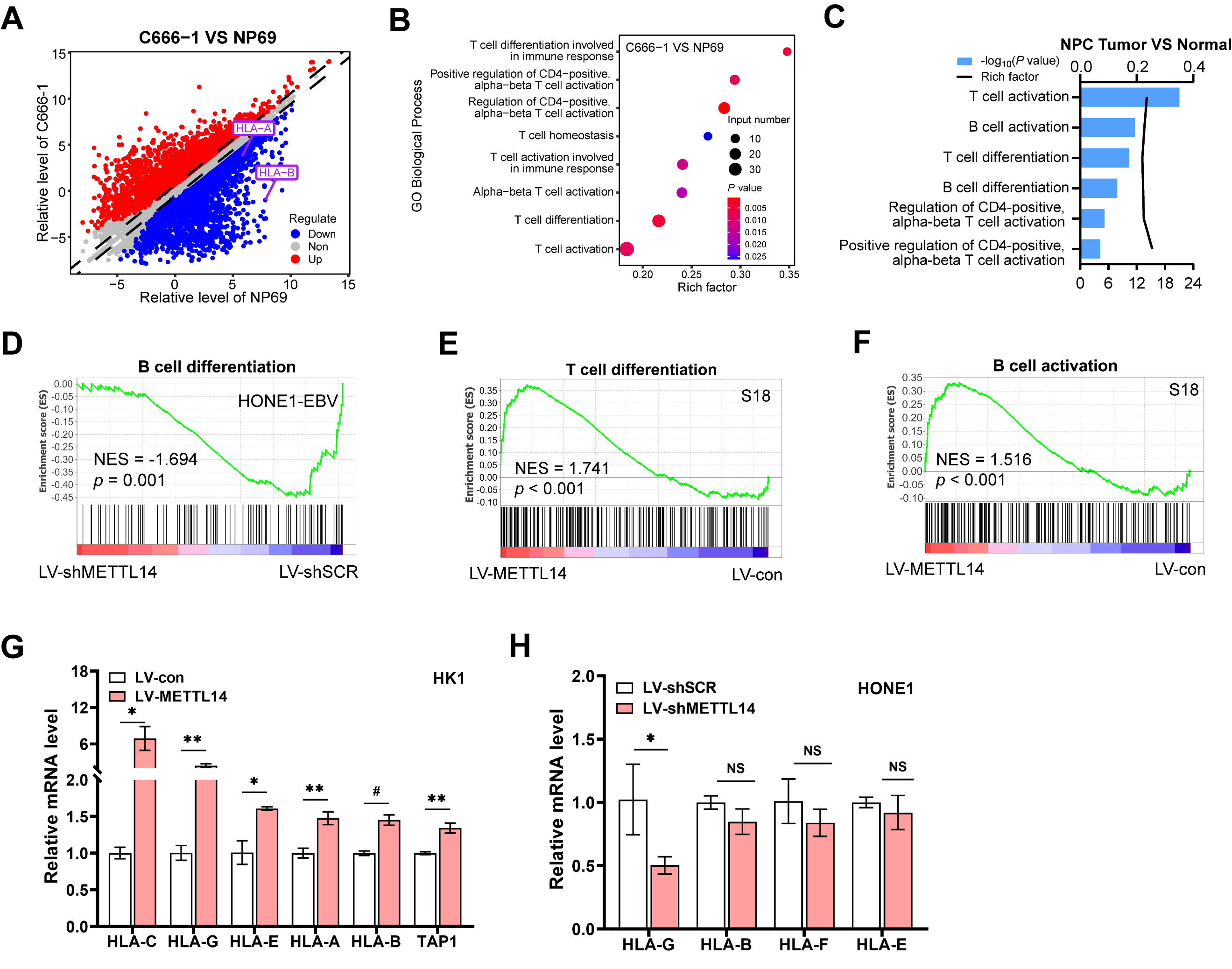


**Supplementary figure 7. MHC-related gene expression in NPC cells and tumors, and GSEA Analysis of MHC-related genes in METTL14- or shMETTL14-expressing NPC cells.**

**(A)** Scatter plots showing the effect of MHC class I genes expression in C666-1 cells and NP69 cells.

**(B)** GO analysis of MHC class Ⅰ genes related up- and down regulated in C666-1 cells and NP69 cells.

**(C)** The histogram of enriched GO terms summarizing the MHC class I genes in up-regulated DEGs in NPC tumors and normal tissues.

**(D–E)** GSEA plot showing enrichment of MHC-related genes expressed between vector-expressing and METTL14-expressing S18 cells.

**(F)** GSEA plot of MHC-related genes enriched in shSCR- and shMETTL14-expressing HONE1-EBV cells.

**(G–H)** qRT-PCR measurement of MHC-related genes expression in HK1 METTL14-expressing and HONE1 shMETTL14-expressing cells.
